# Effects of different types of intermittent fasting on metabolic outcomes: an umbrella review and network meta-analysis

**DOI:** 10.1186/s12916-024-03716-1

**Published:** 2024-11-13

**Authors:** Yu-En Chen, Hui-Li Tsai, Yu-Kang Tu, Ling-Wei Chen

**Affiliations:** 1 Institute of Epidemiology and Preventive Medicine, College of Public Health, National Taiwan University, No. 17 Xu-Zhou Road, Taipei, 100 Taiwan; 2 Institute of Health Data Analytics and Statistics, College of Public Health, National Taiwan University, No. 17 Xu-Zhou Road, Taipei, 100 Taiwan; 3 Master of Public Health Degree Program, College of Public Health, National Taiwan University, No. 17 Xu-Zhou Road, Taipei, 100 Taiwan; 4 Health Data Research Center, National Taiwan University, No.33 Linsen South Road, Taipei, 100 Taiwan

**Keywords:** Intermittent fasting, Alternate-day fasting, Time-restricted eating, Metabolic health, Continuous energy restriction

## Abstract

**Background:**

Intermittent fasting (IF) holds promise for enhancing metabolic health. However, the optimum IF forms and their superiority over continuous energy restriction (CER) remain unclear due to disconnected findings.

**Methods:**

We systematically searched PubMed, Embase, and the Cochrane databases for meta-analyses of randomized controlled trials (RCTs) investigating the association between IF and metabolic health outcomes. Subsequently, we performed an umbrella review and network meta-analysis (NMA) to evaluate the efficacy of different forms of IF (time-restricted eating (TRE), alternate-day fasting (ADF), and 5:2 diet (regular eating for 5 days and energy restriction for 2 days per week)) compared to CER and usual diets on metabolic health outcomes. To assess the certainty of both direct and indirect estimates, we employed the Confidence in Network Meta-Analysis (CINeMA) approach. Additionally, we calculated the surface under the cumulative ranking curve (SUCRA) for each dietary strategy to determine their ranking in terms of metabolic health benefits.

**Results:**

Ten of the best and non-redundant meta-analysis studies, involving 153 original studies and 9846 participants, were included. When considering direct evidence only, all IF forms significantly reduced body weight compared to usual diets. In NMA incorporating indirect evidence, all IF regimens also significantly reduced body weight compared to usual diets. In the SUCRA of NMA, IF ranked higher than usual diets or CER in 85.4% and 56.1% of the outcomes, respectively. ADF had the highest overall ranking for improving metabolic health (ranked first: 64.3%, ranked second: 14.3%).

**Conclusions:**

Overall, all IF forms demonstrate potentials to improve metabolic health, with ADF appearing to produce better outcomes across investigated outcomes. Further high-quality trials are warranted to confirm the (relative) efficacy of IF on metabolic health.

**Trial registration:**

PROSPERO (record no: CRD42022302690).

**Supplementary Information:**

The online version contains supplementary material available at 10.1186/s12916-024-03716-1.

## Background

The widespread occurrence of metabolic syndrome, ranging from 12.5 to 31.4% depending on the region, poses a significant health risk [1], leading to heightened risks of type 2 diabetes [2], cardiovascular diseases [3], non-alcoholic fatty liver disease [4], reproductive system disorders [5], and certain types of cancers [6, 7]. Therefore, addressing metabolic health is a global priority.

Multiple lines of evidence suggest that optimizing lifestyle factors, particularly dietary patterns, can effectively promote metabolic well-being [8–11]. Continuous energy restriction (CER), which aims to reduce overall energy intake per day, is a common dietary strategy for weight loss and subsequent improvement in metabolic parameters. However, CER can be challenging for many individuals to maintain [12, 13]. An alternative dietary approach, known as intermittent fasting (IF), has gained attention. IF is an eating pattern in which individuals alternate between periods of eating and defined extended fasting phases, which may span several hours to days, with energy intake during non-fasting periods either remaining normal or reduced [14]. Common IF regimens include (1) the 5:2 diet, which involves adopting a very low-energy diet for 2 days each week while eating without restrictions for the remaining 5 days [15]; (2) alternate-day fasting (ADF), which entails alternating between very low-energy intake and unrestricted eating every 24 h [14]; and (3) time-restricted eating (TRE), in which individuals typically fast for 14 to 16 h daily, with caloric intake not necessarily restricted [13].

While several meta-analyses have examined the effects of IF compared to CER or usual diets on metabolic health, limited research has investigated the relative efficacy of specific IF formats (5:2, ADF, TRE) and CER in enhancing metabolic parameters. To address this gap, we conducted an umbrella review and utilized network meta-analysis (NMA) to compare the effects of different IF forms and CER on metabolic health.

## Methods

This study was pre-registered with PROSPERO (Registration No.: CRD42022302690). We conducted searches on PubMed, Embase, and Cochrane from their inception to November 29, 2022, using synonyms of IF (see Additional File 1: Table S1 for the detailed search strategy).

### Eligibility criteria

We included meta-analyses of randomized controlled trial (RCT) investigating the association between IF and metabolic health outcomes in humans (details in Additional File 1: Table S2). We excluded the study that (1) was not a meta-analysis, (2) did not investigate IF in relation to metabolic health in humans, and (3) only pooled estimates from an observational design.

### Study screening, data extraction, and the best study selection

Two reviewers, Y.-E.C. and H.-L.T., independently screened articles and extracted data. In cases where disagreements could not be resolved through consensus, a third researcher, L.-W.C., acted as an arbitrator during the negotiation process. For each eligible article, we extracted effect estimates for outcome measures related to metabolic health, including anthropometry measurements, blood pressure, glycemic parameters, and lipid profile.

To prevent duplication of original papers across multiple meta-analyses, we employed a selection process to identify the “best study” for each unique comparison of exposure and outcome [16, 17] based on several criteria: (1) largest number of original studies, (2) results from the Assessment of Multiple Systematic Reviews 2 (AMSTAR2) tool, and (3) largest number of participants in the same comparisons (see Additional File 1: Text S1 for the details of the study selection process) [16, 17].

### Assessment of methodological quality and rating evidence quality

We employed the AMSTAR2 tool [18, 19] to evaluate the methodological quality of all included meta-analyses [14, 15, 20–56] and the established Grading of Recommendations, Assessment, Development and Evaluation (GRADE) approach (criteria and results provided in Additional File 1: Table S3) [37, 57–64] to evaluate the evidence quality for each outcome comparison within the selected best studies [21, 25, 26, 33, 37, 50, 53–56]. Based on these assessments, we classified the result into categories of high, moderate, low, or critically low (very low) quality/certainty.

The Cochrane Risk of Bias tool (RoB 2.0) was used to evaluate the RCT studies included in the NMA, categorizing each study’s overall risk of bias as high, some, or low [65].

Finally, we applied the Confidence in Network Meta-Analysis (CINeMA) framework to assess the confidence in the NMA results and assigned an overall confidence rating of very low, low, moderate, or high (see Additional File 1: Table S4) [61, 66–83].

### Statistical methods

We extracted mean changes, standard deviations (SDs), and sample sizes from the original studies included in the best study. We converted 95% confidence intervals (CIs), standard errors, or interquartile ranges into SDs using the methods described in the Cochrane Handbook [84]. Subsequently, we performed random-effects NMA [85] on each outcome separately using the Stata software (version 16, Stata, College Station, TX, USA). We used the contrast-based model to conduct NMA, and estimated the ranking of different diets in terms of their efficacy on metabolic parameters using the SUCRA (surface under the cumulative ranking curve) analysis. We employed meta-regressions to assess interactions between IF and baseline BMI, age, gender, and intervention duration, using likelihood ratio tests. Sensitivity analyses were conducted by restricting the NMA analyses in healthy populations.

### Summarizing the effectiveness of different dietary strategies on metabolic outcomes

For direct evidence, we organized all pairwise comparisons of IF with usual or CER diets, and color-coded them based on the certainty of our GRADE assessment.

In NMA, which incorporates indirect evidence, we categorized the certainty of the results based on the CINeMA assessment into two groups: (1) moderate to high certainty and (2) very low to low certainty. We then examined the impacts of dietary interventions on metabolic health. If a dietary intervention was significantly better than the reference diet (usual or CER diet) and any other non-reference diet (that was also significantly better than the reference diet), we classified it as having a “major effect” on metabolic health. However, if the dietary intervention was only significantly better than the reference diet, we classified it as having a “minor effect”. If a dietary intervention is not significantly different from the reference diet, it was categorized as having “no effect” [10].

## Results

**Fig. 1:**
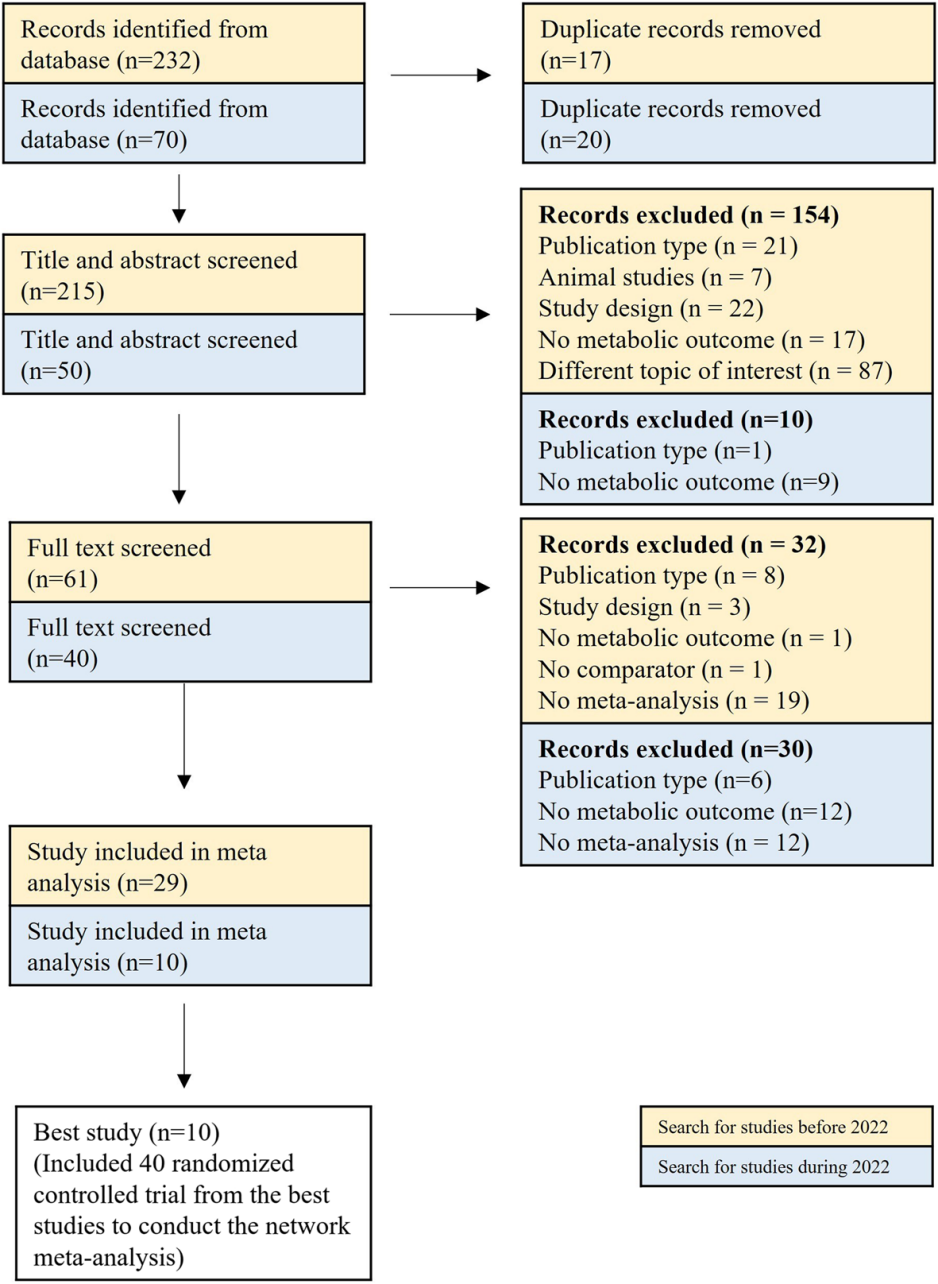
Flowchart for study selection

Out of the initial 302 articles identified across three online databases, 39 were included after a thorough selection process (Fig. 1). To avoid redundancy on the same research question, we further selected 10 best meta-analyses for each unique comparison, as mentioned earlier.

### Study characteristics

Of the 10 best meta-analyses included, five were published in 2022 [50, 53–56]. Each encompassed a range of original studies, ranging from 5 to 43, conducted in countries in North and South America, Asia, Europe, Africa, and Australia/Oceania. The number of included participants in these studies ranged from 131 to 2483, with ages spanning 18 and 79 years old. While three of the meta-analysis studies focused on overweight or obese participants, the others did not impose any weight restrictions. The types of IF examined in these studies included TRE [21, 26, 50, 54, 55], ADF [25, 26, 53–55], 5:2/4:3 diet (days with usual diet: days with very low-energy diet per week) [37, 54, 56], and short-term intermittent energy restriction (STIER) (weekly intermittent energy restriction; fasting lasting more than 7 days) [33]. The duration of the dietary intervention ranged from 2 weeks to 24 months. When assessing methodological quality using AMSTAR2, five studies were rated as critically low quality [21, 25, 26, 33, 53], and the remaining five studies were rated as low quality [37, 50, 54–56] (Table 1).

**Table 1:** Characteristics of included studies

First author (year)	Search start and end dates	No. of included studies	Country of original studies	Total no. of participants	Age (year)	Population	Type of IF	Type of comparator	Diet Duration (months)	AMSTAR2	Unique comparisons and available outcome measurements	
											Comparisons	Outcomes
Harris (2018) [33]	Database inception~ 10/2016	5	Canada, Sweden, USA	376	21~69	Overweight / obese adults	STIER	CER	3.5~12	Critically low quality	[STIER] - [CER]	BW
Cui (2020) [25]	Database inception~ 3/1/2020	7	USA, Korea, Canada, Malaysia, France	269	18~70	Healthy / overweight / obese adults	ADF	Usual diet	1~12	Critically low quality	[ADF] - [Usual]	BW, FM, SBP, DBP, FBG, TC, LDL-C, HDL-C, TG
Park (2020 [26]	Database inception~ ~12/31/2019	8	USA, Austria, Korea, China, UK, Iran, Australia	728	18~65	Healthy / overweight / obese adults	ADF, TRE	CER, usual diet	1~6	Critically low quality	[TRE] - [ADF]	BW, BMI, FM, WC, FBG, TG, TC, LDL-C, HDL-C
											[ADF] - [Usual]	BMI, WC, FI
											[ADF] - [CER]	FI, TC, TG, FBG
Pellegrini (2020) [21]	Database inception~ 1/31/2019	5	USA, Iran, Turkey, Italy, Canada, Denmark, UK	131	22~56 (mean)	Healthy / overweight / obese adults, one with prediabetes	TRE	Usual diet	1~2	Critically low quality	[TRE] - [Usual]	BW, FM, FFM, SBP, DBP, FBG, FI, TG, TC, LDL-C, HDL-C
Schwingshackl (2021) [37]	Database inception~ 3/25/2019	17	USA, Australia, UK, Serbia, Germany, Norway, Malaysia	1328	32~68 (mean)	Healthy / overweight / obese adults, some with MetS, T2DM	5:2; 4:3	CER, usual diet	3~13	Low quality	[5:2/4:3] - [Usual]	BW, FM, WC, SBP, FBG, HbA1c, LDL-C, TG
											[5:2/4:3] - [CER]	BW, FM, WC, SBP, FBG, HbA1c, LDL-C, TG
Elortegui Pascual (2022) [53]	Database inception~ 6/7/2021	24	NA^a^	1768	23~68 (mean)	Healthy / overweight / obese adults, without MetS, prediabetes, or T2DM	ADF, 5:2, TRE	CER, usual diet	0.5~6.5	Critically low quality	[ADF] - [CER]	BW
Gu (2022) [55]	Database inception~ 6/2021	43	Brazil, China, Germany, Iran, Italy, Korea, Malaysia, New Zealand, Norway, Spain, Tunisia, Turkey, the UK, USA	2483	18~79	Healthy / overweight / obese adults	IER, 5:2, ADF, TRE, RDIF	CER, usual diet	1~16	Low quality	[TRE] - [CER]	BW, BMI, FM, FFM, WC, SBP, DBP
											[ADF] - [CER]	BMI, FM, WC, SBP
Kim (2022) [54]	2011~ 12/31/2021	16	NA^a^	1074	18~70	Overweight / obese adults, some with MetS, T2DM	ADF, 5:2, TRE	CER	3~13	Low quality	[5:2] - [CER]	BW, BMI, WC, SBP, DBP, FBG, FI, HbA1c, TC, LDL-C, HDL-C, TG
											[ADF] - [CER]	FFM, DBP, LDL-C, HDL-C
											[TRE] - [CER]	FBG, FI, HbA1c, TC, LDL-C, HDL-C, TG
Liu (2022) [50]	Database inception~ 2/26/2022	17	China, Switzerland, USA, Germany, Brazil, Italy	899	19~54 (mean)	Healthy / overweight / obese adults, some with MetS, NAFLD, T2DM	TRE	CER, usual diet	1~12	Low quality	[TRE] - [Usual/CER]	BW, BMI, FM, WC, SBP, DBP, FBG, HbA1c, TC, LDL-C, HDL-C, TG
Wang (2022) [56]	Database inception~ 12/2021	11	NA^a^	790	18~75	Overweight / obese adults, some with MetS, T2DM	5:2, ADF	CER	1~24	Low quality	[5:2] - [CER]	FM, FFM

*4:3* Regular eating for four days and energy restriction for three days per week, *5:2 *Regular eating for five days and energy restriction for two days per week, *ADF* Alternate day fasting, *BMI *Body mass index, *BW *Body weight, *CER *Continuous energy restriction, *DBP *Diastolic blood pressure, *FBG *Fasting blood glucose, *FFM *Fat free mass, *FI *Fasting insulin, *FM *Fat mass, *HbA1c *Glycosylated hemoglobin, *HDL-C *High density lipoprotein cholesterol, *IF *Intermittent fasting, *LDL-C *Low density lipoprotein cholesterol, *MetS *Metabolic syndrome, *NAFLD *Non-alcoholic fatty liver disease, *RDIF *Ramadan diurnal intermittent fasting, *SBP *Systolic blood pressure, *STIER *Short-term intermittent energy restriction, *T2DM *Type 2 Diabetes Mellitus, *TC *Total cholesterol, *TG *Triglycerides, *TRE *Time-restricted eating, *UK *United Kingdom, *USA *United States of America, *WC *Waist circumference

^a^The systematic review did not include information about the countries where the original studies were conducted

### Effects of IF on metabolic health: a qualitative analysis of direct evidence

The summary of the comparison of different dietary interventions is presented in Table 2, and the forest plot is displayed in Additional File 1: Fig. S1.

**Table 2:** Summary of the direct evidence on effects of IF on metabolic outcomes in included meta-analyses

Comparison		No. of studies	No. of population	Population characteristics				Diet duration (months)	Mean difference (95% CI)	*I*^*2*^	AMSTAR2	GRADE
IF form	Reference diet			BMI			Disease					
				Normal	Overweight	Obesity						
**Anthropometric measures**												
**BW, kg**												
TRE	Usual	5	131	**✓**	**✓**	**✓**	preDM	1~2	**-0.38 (-0.71, -0.05)**	0	Critically low	Low
ADF		7	269	**✓**	**✓**	**✓**	-	1~12	**-4.30 (-5.54, -3.05)**	96	Critically low	Very low
5:2/4:3		6	348	**✓**	**✓**	**✓**	-	3~6	**-4.83 (-5.46, -4.21)**	3	Low	Low
TRE	CER	8	-	**✓**	**✓**	**✓**	-	1~16	-2.41 (-5.14, 0.33)	42	Low	Moderate
ADF		8	393	**✓**	**✓**	**✓**	-	0.75~6.5	-0.17 (-1.03, 0.69)	76.2	Critically low	Very low
5:2		8	700	-	**✓**	**✓**	-	3~13	-0.02 (-0.17, 0.13)	0	Low	High
5:2/4:3		13	991	**✓**	**✓**	**✓**	T2DM	3~13	**-0.55 (-1.01, -0.09)**	0	Low	Moderate
STIER		5	317	-	**✓**	**✓**	-	3.5~12	-1.36 (-3.23, 0.51)	37.6	Critically low	Low
TRE	Usual/CER	17	899	**✓**	**✓**	**✓**	MetS, NAFLD, T2DM	1.3~12	**-1.60 (-2.27, -0.93)**	90.2	Low	Low
TRE	ADF	1	185	-	**✓**	**✓**	-	3	0.39 (-1.68, 2.46)	-	Critically low	Very low
**BMI, kg/m**^2^												
ADF	Usual	4	293	**✓**	**✓**	**✓**	-	1~3	**-0.96 (-1.42, -0.50)**	52	Critically low	Low
TRE	CER	4	-	**✓**	**✓**	**✓**	-	1~16	-0.66 (-2.22, 0.91)	51	Low	Moderate
ADF		5	-	**✓**	**✓**	**✓**	-	1~16	-0.66 (-1.57, 0.24)	45	Low	Moderate
5:2		5	436	-	**✓**	**✓**	T2DM	3~13	0.08 (-0.11, 0.27)	0	Low	High
TRE	Usual/CER	8	616	**✓**	**✓**	**✓**	MetS, NAFLD, T2DM	2~12	-0.53 (-1.10, 0.04)	84.5	Low	Low
TRE	ADF	1	185	-	**✓**	**✓**	-	3	**0.45 (0.01, 0.89)**	-	Critically low	Very low
**FM, kg**												
TRE	Usual	4	100	**✓**	-	-	-	1.3~2	-0.83 (-1.89, 0.24)	31	Critically low	Low
ADF		6	226	**✓**	**✓**	**✓**	-	1~12	-4.96 (-8.08, 1.85)	99	Critically low	Very low
5:2/4:3		6	279	**✓**	**✓**	**✓**	-	3~6	**-2.54 (-3.78, -1.31)**	62	Low	Low
TRE	CER	5	-	**✓**	**✓**	**✓**	-	1~16	-2.58 (-4.55, 0.61)	45	Low	Moderate
ADF		6	-	**✓**	**✓**	**✓**	-	1~16	-2.80 (-6.34, 0.74)	78	Low	Low
5:2		5	438	**-**	**✓**	**✓**	T2DM	3~24	0.06 (-1.02, 1.14)	0	Low	High
5:2/4:3		10	743	**-**	**✓**	**✓**	T2DM	3~13	**-0.66 (-1.14, -0.19)**	0	Low	High
TRE	Usual/CER	13	629	**✓**	**✓**	**✓**	NAFLD	1.3~12	**-1.48 (-1.59, -1.38)**	32	Low	High
TRE	ADF	1	185	**✓**	**✓**	**✓**	-	3	0.55 (-0.30, 1.40)	-	Critically low	Very low
**FFM, kg**												
TRE	Usual	4	100	**✓**	**-**	**-**	**-**	1.5~2	0.00 (-0.78, 0.79)	0	Critically low	Low
TRE	CER	5	-	**✓**	**✓**	**✓**	**-**	1~16	-1.27 (-2.82, 0.29)	2	Low	High
ADF		5	342	**-**	**✓**	**✓**	**-**	3~6.5	0.08 (-0.13, 0.30)	0	Low	Moderate
5:2		5	438	**-**	**✓**	**✓**	T2DM	1~24	-0.23 (-0.97, 0.51)	29	Low	High
**WC, cm**												
ADF	Usual	2	201	**-**	**✓**	**✓**	**-**	3	-2.41 (-4.95, 0.13)	63.1	Critically low	Low
5:2/4:3		2	142	**-**	**✓**	**✓**	**-**	3	-1.73 (-3.69, 0.24)	0	Low	Moderate
TRE	CER	6	-	**✓**	**✓**	**✓**	**-**	1~16	-3.66 (-6.08, 1.24)	38	Low	High
ADF		3	-	**✓**	**✓**	**✓**	-	1~16	-1.37 (-4.08, 1.34)	28	Low	High
5:2		5	393	**-**	**✓**	**✓**	-	3~6.5	-0.06 (-0.26, 0.14)	0	Low	Moderate
5:2/4:3		8	641	**-**	**✓**	**✓**	T2DM	3~10.5	-0.57 (-1.56, 0.41)	0	Low	High
TRE	Usual/CER	7	512	**✓**	**✓**	**✓**	MetS, NAFLD	2~12	-0.07 (-0.98, 0.83)	0	Low	High
TRE	ADF	1	185	**-**	**✓**	**✓**		3	0.39 (-0.95, 1.73)	-	Critically low	Very low
**Blood pressure**												
**SBP, mmHg**												
TRE	Usual	2	33	**✓**	-	-	-	2	-2.27 (-19.52, 14.98)	88.9	Critically low	Very low
ADF		4	175	**✓**	**✓**	**✓**	-	1~12	**-4.42 (-7.35, -1.49)**	84	Critically low	Very low
5:2/4:3		5	254	**✓**	**✓**	**✓**	-	3	**-6.11 (-9.59, -2.64)**	0	Low	Moderate
TRE	CER	4	-	**✓**	**✓**	**✓**	-	1~16	-3.19 (-7.63, 1.26)	0	Low	High
ADF		3	-	**✓**	**✓**	**✓**	-	1~16	-4.34 (-14.50, 5.83)	79	Low	Low
5:2		4	301	-	**✓**	**✓**	-	3~6.5	0.20 (-0.03, 0.42)	0	Low	Moderate
5:2/4:3		8	572	-	**✓**	**✓**	T2DM	3~6.5	0.24 (-2.08, 2.55)	0	Low	High
TRE	Usual/CER	8	454	**✓**	**✓**	**✓**	MetS	1.3~12	-2.46 (-6.43, 1.51)	77.4	Low	Low
**DBP, mmHg**												
TRE	Usual	2	33	**✓**	**-**	**-**	-	2	-2.76 (-19.52, 14.98)	88.1	Critically low	Very low
ADF		4	175	**✓**	**✓**	**✓**	-	1~12	**-3.41 (-5.91, -0.92)**	80	Critically low	Very low
TRE	CER	4	-	**✓**	**✓**	**✓**	-	1~16	-3.47 (-7.00, 0.06)	0	Low	Moderate
ADF		3	239	**-**	**✓**	**✓**	-	3~6.5	-0.04 (-0.29, 0.22)	0	Low	Moderate
5:2		3	224	**-**	**✓**	**✓**	-	3~6.5	-0.04 (-0.30, 0.22)	0	Low	Moderate
TRE	Usual/CER	8	454	**✓**	**✓**	**✓**	MetS	1.3~12	-1.78 (-4.72, 1.16)	72.2	Low	Low
**Glycemic parameters**												
**FBG, mg/dl**												
TRE	Usual	4	90	**✓**	**✓**	**✓**	preDM	1.3~2	**-2.45 (-4.72, -0.17)**	0	Critically low	Low
ADF		4	144	**✓**	**✓**	**✓**	-	2~6	-3.02 (-6.52, 0.48)	89	Critically low	Very low
5:2/4:3		4	224	**-**	**✓**	**✓**	-	3	-2.16 (-7.38, 3.24)	43	Low	Low
TRE	CER	1	65	**-**	**✓**	**✓**	MetS	3	-0.09 (-0.57, 0.40)	-	Low	Very low
ADF		3	225	**✓**	**✓**	**✓**	-	1~6	-1.34 (-4.74, 2.06)	55.6	Critically low	Low
5:2		7	545	**-**	**✓**	**✓**	-	3~13	0.15 (-0.02, 0.33)	89	Low	Low
5:2/4:3		8	584	**-**	**✓**	**✓**	T2DM	3~10.5	1.08 (-0.54, 2.70)	5	Low	High
TRE	Usual/CER	12	676	**✓**	**✓**	**✓**	NAFLD, T2DM	1~6	**-4.08 (-7.74, -0.42)**	96.6	Low	Low
TRE	ADF	1	185	**-**	**✓**	**✓**	-	3	-2.52 (-8.01, 2.97)	-	Critically low	Very low
**FI, μIU/ml**												
TRE	Usual	4	90	**✓**	**✓**	**✓**	preDM	1.3~2	-0.69 (-1.64, 0.26)	48.9	Critically low	Very low
ADF		2	67	**-**	**✓**	**✓**	-	2~3	-1.50 (-10.11, 7.11)	0	Critically low	Moderate
TRE	CER	1	65	**-**	**✓**	**✓**	MetS	3	0.02 (-0.46, 0.51)	-	Low	Very low
ADF		3	226	**-**	**✓**	**✓**	-	2~6	0.12 (-2.34, 2.59)	55.8	Critically low	Low
5:2		4	315	**-**	**✓**	**✓**	-	3~13	-0.09 (-0.31, 0.14)	81	Low	Very low
**HbA1c, %**												
5:2/4:3	Usual	1	101	**-**	**✓**	**✓**	**-**	3	0.00 (-0.08, 0.08)	-	Low	Very low
TRE	CER	1	65	**-**	**✓**	**✓**	MetS	3	-0.01 (-0.50, 0.47)	-	Low	Very low
5:2		4	388	**-**	**✓**	**✓**	GDM, T2DM	3~13	0.01 (-0.19, 0.21)	65	Low	Moderate
5:2/4:3		6	570	**-**	**✓**	**✓**	MetS	3~13	-0.01 (-0.07, 0.06)	0	Low	High
TRE	Usual/CER	6	355	**-**	**✓**	**✓**	MetS, T2DM	2~6	-0.11 (-0.50, 0.27)	98.8	Low	Very low
**Lipid profile**												
**TG, mg/dl**												
TRE	Usual	4	90	**✓**	**✓**	**✓**	preDM	1.3~2	6.24 (-13.40, 25.88)	86.8	Critically low	Very low
ADF		5	208	**✓**	**✓**	**✓**	-	2~12	**-11.27 (-20.53, -2.00)**	96	Critically low	Very low
5:2/4:3		5	254	**✓**	**✓**	**✓**	-	3	**-17.71 (-33.66, -2.66)**	52	Low	Low
TRE	CER	1	65	**-**	**✓**	**✓**	MetS	3	-0.02 (-0.51, 0.47)	-	Low	Very low
ADF		3	225	**✓**	**✓**	**✓**	-	2~6	-5.87 (-32.06, 20.32)	50.5	Critically low	Low
5:2		6	494	**✓**	**✓**	**✓**	-	3~13	0.06 (-0.12, 0.24)	25	Low	High
5:2/4:3		8	587	**-**	**✓**	**✓**	T2DM	3~10.5	-3.54 (-11.51, 5.31)	0	Low	High
TRE	Usual/CER	13	767	**✓**	**✓**	**✓**	MetS, NAFLD, T2DM	1.3~12	-8.64 (-18.01, 0.73)	97	Low	Low
TRE	ADF	1	185	**-**	**✓**	**✓**	-	3	16.81 (-29.84, 63.47)	-	Critically low	Very low
**TC, mg/dl**												
TRE	Usual	4	90	**✓**	**✓**	**✓**	preDM	1.3~2	9.14 (-3.69, 21.96)	80.9	Critically low	Very low
ADF		5	174	**✓**	**✓**	**✓**	-	2~12	**-11.32 (-18.20, -4.44)**	96	Critically low	Very low
TRE	CER	1	65	**-**	**✓**	**✓**	MetS	3	0.00 (-0.48, 0.49)	-	Low	Very low
ADF		3	225	**-**	**✓**	**✓**	-	2~6	-2.79 (-11.31, 5.74)	0	Critically low	Moderate
5:2		6	494	**-**	**✓**	**✓**	-	3~13	**-0.2 (-0.37, -0.02)**	51	Low	Low
TRE	Usual/CER	10	657	**✓**	**✓**	**✓**	NAFLD, T2DM	1~12	**-6.10 (-7.86, -4.34)**	4.6	Low	High
TRE	ADF	1	185	**-**	**✓**	**✓**	-	3	8.49 (-6.09, 23.08)	-	Critically low	Very low
**LDL-C, mg/dl**												
TRE	Usual	4	90	**✓**	**✓**	**✓**	preDM	1.3~2	3.99 (-6.41, 14.39)	69.3	Critically low	Very low
ADF		4	151	**✓**	**✓**	**✓**	-	2~12	**-5.82 (-10.45, -1.19)**	92	Critically low	Very low
5:2/4:3		5	254	**✓**	**✓**	**✓**	-	3	-1.44 (-5.40, 2.70)	67	Low	Very low
TRE	CER	1	65	**-**	**✓**	**✓**	MetS	3	-0.07 (-0.55, 0.42)	-	Low	Very low
ADF		3	229	**-**	**✓**	**✓**	**-**	3~6.5	0.00 (-0.26, 0.26)	0	Low	Moderate
5:2		6	494	**-**	**✓**	**✓**	-	3~13	**-0.20 (-0.38, -0.02)**	0	Low	High
5:2/4:3		8	579	**-**	**✓**	**✓**	T2DM	3~10.5	0.00 (-1.98, 1.98)	19	Critically low	Moderate
TRE	Usual/CER	10	689	**✓**	**✓**	**✓**	NAFLD, T2DM	1.3~12	-3.63 (-8.05, 0.78)	81.3	Low	Low
TRE	ADF	1	185	**-**	**✓**	**✓**	-	3	-3.09 (-12.55, 6.37)	-	Critically low	Very low
**HDL-C, mg/dl**												
TRE	Usual	4	90	**✓**	**✓**	**✓**	preDM	1.3~2	1.36 (-1.43, 4.14)	53.9	Critically low	Very low
ADF		5	174	**✓**	**✓**	**✓**	-	2~12	-1.05 (-2.92, 0.83)	71	Critically low	Low
TRE	CER	1	65	**-**	**✓**	**✓**	MetS	3	0.18 (-0.31, 0.66)	-	Low	Very low
ADF		3	229	**-**	**✓**	**✓**	-	3~6.5	**-0.34 (-0.61, -0.08)**	79	Low	Moderate
5:2		6	494	**-**	**✓**	**✓**	-	3~13	0.05 (-0.12, 0.23)	0	Low	High
TRE	Usual/CER	12	755	**✓**	**✓**	**✓**	NAFLD, T2DM	1~12	0.75 (-0.73, 2.24)	73.6	Low	Moderate
TRE	ADF	1	185	**-**	**✓**	**✓**	-	3	-0.77 (-5.40, 3.85)	-	Critically low	Very low

Bold font: statistically significant associations

*4:3 *Regular eating for four days and energy restriction for three days per week, *5:2 *Regular eating for five days and energy restriction for two days per week, *ADF *Alternate day fasting, *BMI *Body mass index, *BW *Body weight, *CER *Continuous energy restriction, *DBP *Diastolic blood pressure, *FBG *Fasting blood glucose, *GDM *Gestational diabetes mellitus, *FFM *Fat free mass, *FI *Fasting insulin, *FM *Fat mass, *HbA1c *Hemoglobin A1c, *HDL-C *High density lipoprotein cholesterol, *IF *Intermittent fasting, *LDL-C *Low density lipoprotein cholesterol, *MetS* Metabolic syndrome, *NAFLD *Nonalcoholic fatty liver disease, *preDM *Prediabetes mellitus, *SBP S*ystolic blood pressure, *STIER *Short-term intermittent energy restriction, *T2DM *Type 2 diabetes mellitus, *TC *Total cholesterol, *TG *Triglycerides, *TRE *Time-restricted eating, *WC *Waist circumference

#### Anthropometric measures

There were ten unique meta-analysis comparisons for body weight (BW) [21, 25, 26, 33, 37, 50, 53–55] (Table 2). When comparing IF (TRE [21], ADF [25], 5:2/4:3 [37]) to usual diets, all showed a significant reduction in body weight (range of mean difference: − 0.38 to − 4.83 kg), but with low or very low certainty. When compared to CER, only the 5:2/4:3 diet demonstrated a significant effect on reducing body weight (difference in mean: − 0.55 kg; 95% CI: − 1.01 to − 0.09) with moderate certainty [37]. TRE approach also reduced body weight compared to usual/CER diets (− 1.60 kg; − 2.27 to − 0.93), with low certainty [50].

There were six unique meta-analysis comparisons for body mass index (BMI) [26, 50, 54, 55] (Table 2). When comparing different diets, only ADF demonstrated a significant reduction in BMI compared to usual diets [26] (− 0.96 kg/m^2^; − 1.42 to − 0.50) and TRE (− 0.45 kg/m^2^; − 0.89 to − 0.01) [26], with low and very low certainty [26].

There were nine unique meta-analysis comparisons for fat mass (FM) [21, 25, 26, 37, 50, 55, 56] (Table 2). The 5:2/4:3 diet significantly reduced FM with low (compared to usual diets) (− 2.54 kg; − 3.78 to − 1.31) and high certainty (compared to CER) (− 0.66 kg; − 1.14 to − 0.19) [37]. Additionally, TRE also significantly reduced FM (− 1.48 kg; − 1.59 to − 1.38) compared to usual/CER diets with high certainty [50].

However, no significant differences were observed between any form of IF and the reference diets in fat-free mass (FFM) and waist circumference (WC) outcomes (Table 2).

#### Blood pressure

There were eight unique meta-analysis comparisons for systolic blood pressure (SBP) [21, 25, 37, 50, 54, 55] (Table 2). Only ADF (− 4.42 mmHg; − 7.35 to − 1.49; very low certainty) [25] and 5:2/4:3 diet (− 6.11 mmHg; − 9.59 to − 2.64; moderate certainty) [37] significantly reduced SBP compared to usual diets.

There were six unique meta-analysis comparisons for diastolic blood pressure (DBP) [21, 25, 50, 54, 55] (Table 2). Only ADF significantly reduced DBP compared to usual diets (− 3.41 mmHg; − 5.91 to − 0.92) [25], albeit with very low certainty.

#### Glycemic parameters

There were nine unique meta-analysis comparisons for fasting blood glucose (FBG) [21, 25, 26, 37, 50, 54] (Table 2). Only TRE significantly reduced FBG compared to usual diets (− 2.45 mg/dl; − 4.72 to − 0.17) [21] or usual/CER diets (− 4.08 mg/dl; − 7.74 to − 0.42) [50], with low certainty for both comparisons.

However, no significant differences were observed between any IF form and the reference diets in fasting insulin (FI) and glycosylated hemoglobin (HbA1c) outcomes (Table 2).

#### Lipid profile

There were nine unique meta-analysis comparisons for triglycerides (TG) [21, 25, 26, 37, 50, 54] (Table 2). Only ADF (− 11.27 mg/dl; − 20.53 to − 2.00) [25] and 5:2/4:3 diet (− 17.71 mg/dl; − 33.66 to − 2.66) [37] significantly reduced TG levels compared to usual diets, with very low and low certainty.

There were seven unique meta-analysis comparisons for total cholesterol (TC) [21, 25, 26, 50, 54] (Table 2). TRE significantly reduced TC levels compared to usual/CER diets (− 6.10 mg/dl; − 7.86 to − 4.34) with high certainty [50]. Additionally, the 5:2 diet significantly reduced TC levels compared to CER (− 0.2 mg/dl; − 0.37 to − 0.02) [54] with low certainty and ADF significantly reduced TC levels compared to usual diets (− 11.32 mg/dl; − 18.20 to − 4.44) [25] with very low certainty.

There were nine unique meta-analysis comparisons for low density lipoprotein cholesterol (LDL-C) [21, 25, 26, 37, 50, 54] (Table 2). When compared to CER, only the 5:2 diet significantly reduced LDL-C levels (− 0.20 mg/dl; − 0.38 to − 0.02), with high certainty [54]. ADF significantly reduced LDL-C levels compared to usual diets (− 5.82 mg/dl; − 10.45 to − 1.19) with very low certainty [25].

There were seven unique meta-analysis comparisons for high density lipoprotein cholesterol (HDL-C) [21, 25, 26, 50, 54] (Table 2). Among all comparisons, only the ADF diet significantly reduced HDL-C levels compared to CER (− 0.34 mg/dl; − 0.61 to − 0.08), with moderate certainty [54].

### Summary of the direct evidence with GRADE assessment

**Fig. 2:**
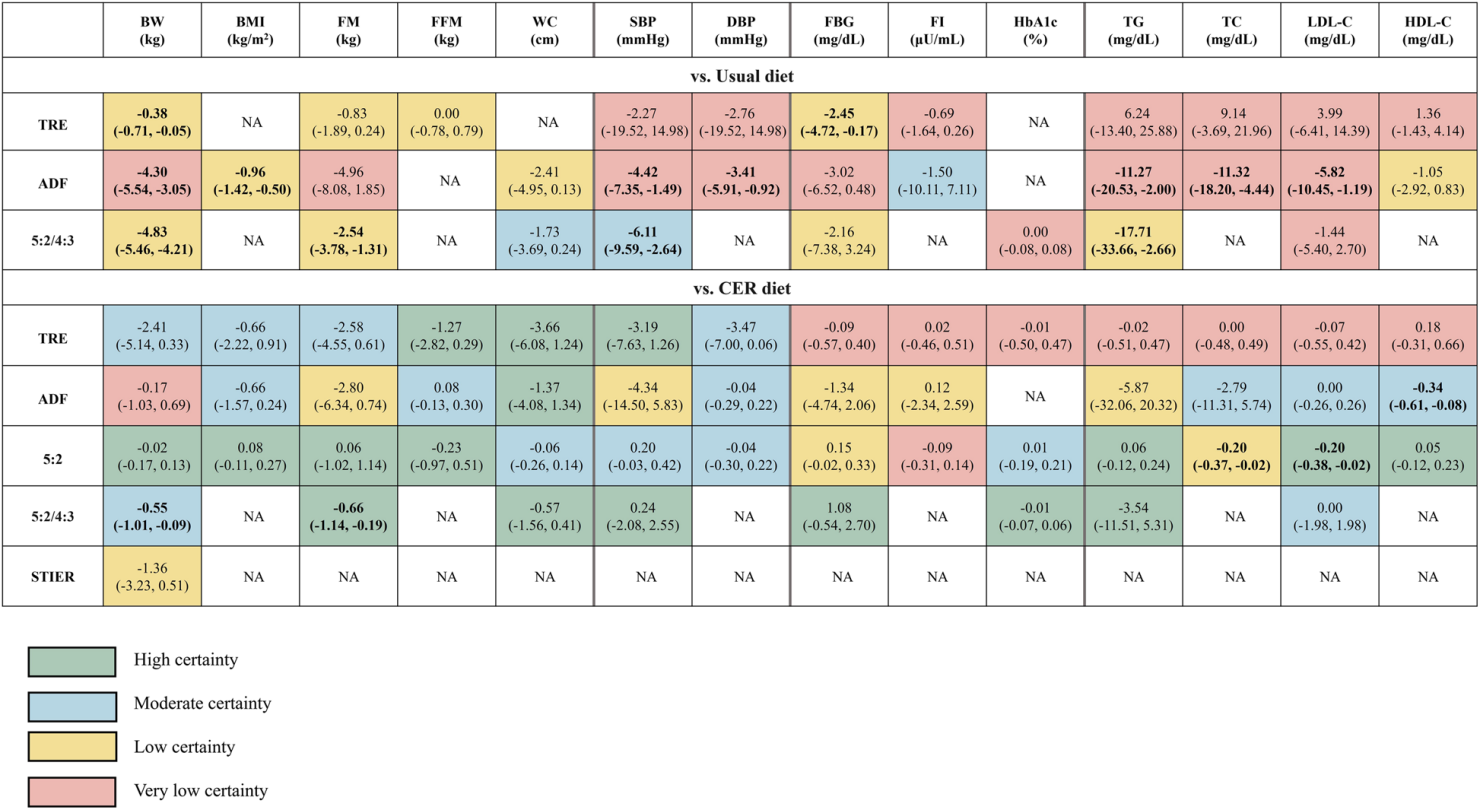
All comparisons of direct evidence. Effect estimates are mean differences (95% CI). Bold font: statistically significant associations. 4:3: regular eating for 4 days and energy restriction for 3 days per week, 5:2: regular eating for 5 days and energy restriction for 2 days per week. ADF, alternate day fasting; BMI, body mass index; BW, body weight; CER, continuous energy restriction; DBP, diastolic blood pressure; FBG, fasting bloodglucose; FFM, fat-free mass; FI, fasting insulin; FM, fat mass; HbA1c, glycosylated hemoglobin A1c; HDL-C, high-density lipoprotein cholesterol; LDL-C, low-density lipoprotein cholesterol; SBP, systolic blood pressure; STIER, short-term intermittent energy restriction; TC, total cholesterol; TG, triglycerides; TRE, time-restricted eating; WC, waist circumference

The summary of the direct evidence with our GRADE assessment is presented in Fig. 2. IF significantly improved metabolic health outcomes in 42% (13 out of 31) of the comparisons with usual diets, with efficacy shown across all IF forms for body weight reduction, albeit with low or very low certainty. Among IF regimens, ADF generally demonstrated superior performance across metabolic health outcomes, but most of the evidence associated with ADF had very low certainty. The only significant association supported by moderate certainty is the reduction in SBP associated with the 5:2/4:3 diet.

When compared to CER, only 8% (4 out of 53) of the comparisons indicated that IF significantly improved metabolic health. Notably, the 5:2 diet performed significantly better than CER for LDL-C levels, while the 5:2/4:3 diet exhibited significant improvements in BW and FM, all with moderate and high certainty.

### Effects of IF on metabolic health: results from quantitative network meta-analysis

Supplementary Additional File 1: Table S5 [86–125] and Fig. S2 presents the characteristics and risk of bias of the original studies included in the best studies, respectively. Additional File 1: Table S4 provides a detailed assessment of the certainty of evidence using the CINeMA approach, while Additional File 1: Table S6 offers network evidence for pairwise comparisons. Additional File 1: Fig. S3 displays the network plots, and Additional File 1: Fig. S4 details the SUCRA. Below, we summarize the key findings based on the criteria outlined in the Methods section.

To determine the specific impact of a specific dietary strategy on metabolic outcomes in the quantitative NMA, we further excluded comparisons involving mixed strategies (5:2/4:3) and STIER (as it deviated from the standard regimen of IF and provided only BW outcome data compared to CER).

#### NMA results for anthropometric measures

**Fig. 3:**
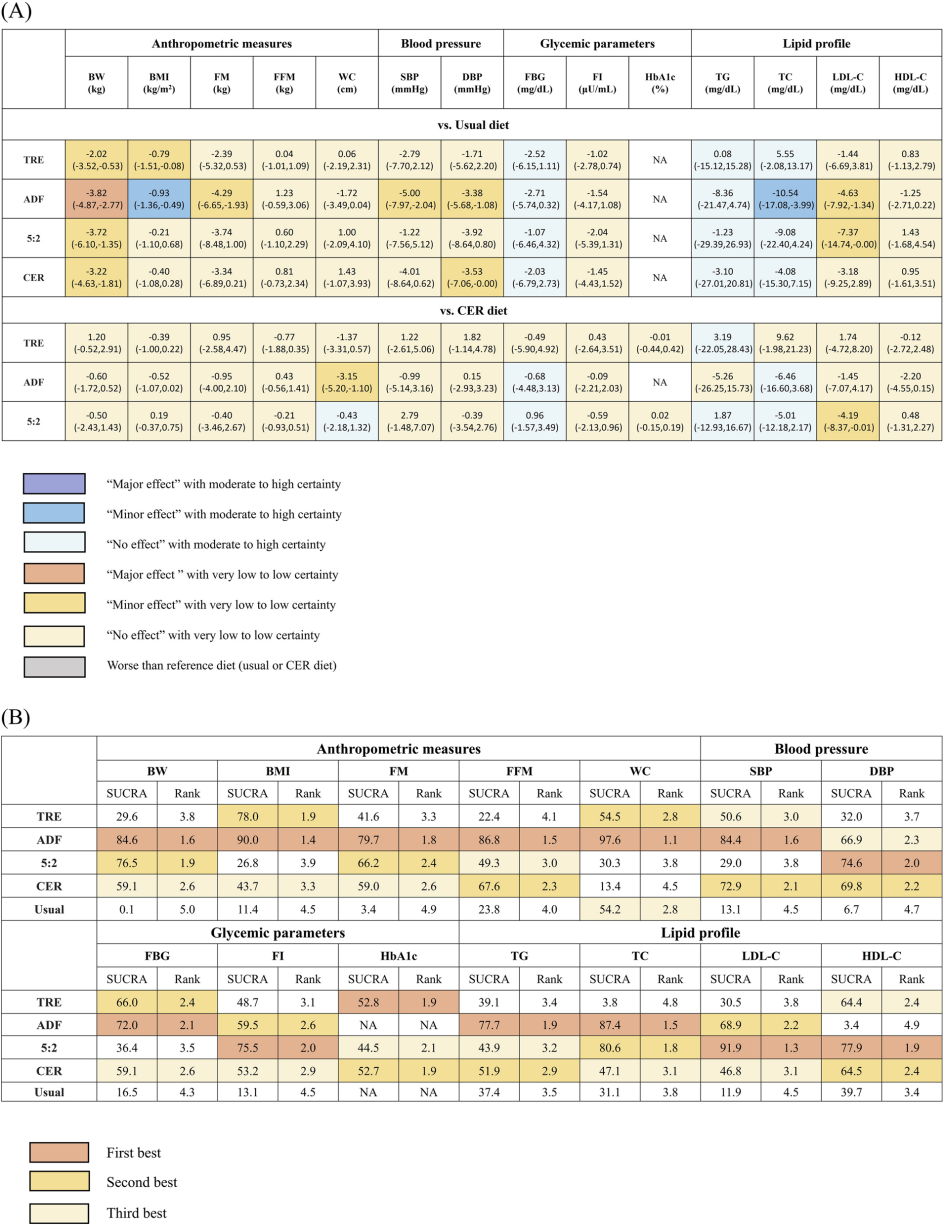
Summary results of network meta-analysis. **A** Different diet compared to usual or CER diet. **B** Ranking by SUCRA analysis. Effect estimates for panel **A** are mean differences (95% CI). Numbers presented in panel **B** are SUCRA values and mean rank; greater SUCRA values or lower mean rank values indicate superior dietary strategies. 5:2: regular eating for 5 days and energy restriction for 2 days per week. ADF, alternate day fasting; BMI, body mass index; BW, body weight; CER, continuous energy restriction; DBP, diastolic blood pressure; FBG, fasting blood sugar; FFM, fat-free mass; FI, fasting insulin; FM, fat mass; HbA1c, glycosylated hemoglobin A1c; HDL-C, high-density lipoprotein cholesterol; LDL-C, low-density lipoprotein cholesterol; SBP, systolic blood pressure; SUCRA, surface under the cumulative ranking curve; TC, total cholesterol; TG, triglycerides; TRE, time-restricted eating; WC, waist circumference. Mixed strategy comparisons (5:2/4:3) and STIER (as it deviated from the standard regimen of IF) were excluded in this quantitative network meta-analysis

When compared to usual diet, ADF showed major effect on BW, albeit with low certainty. TRE and the 5:2 diet demonstrated minor effects on BW, with low-very low certainty. ADF and TRE had minor effects on BMI, with ADF showing moderate certainty and TRE showing very low certainty. ADF exhibited a minor effect on FM, with very low certainty (Fig. 3A).

When compared to CER, only ADF demonstrated minor effects on WC with very low certainty (Fig. 3A).

When compared to TRE, ADF (− 1.79 kg; − 3.38 to − 0.21) significantly decreased BW, albeit with low certainty (Additional File 1: Table S6). According to the SUCRA analysis, ADF consistently ranked first for anthropometric measures (Fig. 3B).

#### NMA results for blood pressure

When compared to usual diets, ADF demonstrated a minor effect on both SBP and DBP with low-very low certainty (Fig. 3A).

However, no significant differences were observed when comparing IF to CER or within the IF group itself (Additional File 1: Table S6). In terms of SUCRA rankings, ADF ranked first and third for SBP and DBP, respectively (Fig. 3B).

#### NMA results for glycemic parameters

When compared to usual diets, all forms of IF showed no significant effect on FBG and FI, with moderate-high certainty for FBG and low-very low certainty for FI.

No significant differences were observed when comparing IF to CER or within the IF group itself (Additional File 1: Table S6). In the SUCRA analysis, ADF ranked first or second for FBG and FI, while the rankings of other IF forms were inconsistent (Fig. 3B).

#### NMA results for lipid profile

When compared to usual diets, ADF had a minor effect on TC and LDL-C with moderate and low certainty, respectively. 5:2 diet also had a minor effect on LDL-C with low certainty.

When compared to CER, 5:2 diet showed a minor effect on LDL-C levels with low certainty. (Fig. 3A).

When compared to TRE, both the 5:2 diet (− 14.63 mg/dl; − 28.27 to − 0.99) with moderate certainty and ADF (− 16.09 mg/dl; − 25.06 to − 7.11) with low certainty significantly decreased TC (Additional File 1: Table S6). Based on SUCRA analysis, ADF ranked first for TG and TC, while the 5:2 diet ranked first for LDL-C and HDL-C (Fig. 3B).

In our network meta-analysis, across all studied outcomes, IF in general ranked better than usual diets (85.4% of the comparisons) and CER (56.1% of the comparisons).

#### Meta-regression and sensitivity analyses

Several significant interactions were identified between IF and baseline BMI, age, gender, and duration of intervention in the NMA meta-regression analyses (Additional File 1: Table S7). Specifically, a higher baseline BMI decreased the treatment effects of both ADF and TRE on BW and FM, of ADF on WC, but increased the treatment effect of the 5:2 diet on WC compared to a usual diet. Longer intervention duration decreased the treatment effects of both ADF and TRE on BW, and of both ADF and the 5:2 diet on FM. Lastly, the treatment effect of ADF on WC decreased with age. However, these results should be interpreted with caution due to the limited number of studies available for each treatment comparison. In sensitivity analyses restricted to healthy populations, ADF still consistently displayed higher rankings for most outcomes (except for glycemic parameters), aligned with the main analyses (Additional File 1: Fig. S5).

## Discussion

To our knowledge, this umbrella review is the first to comprehensively elucidate the impact of IF diets on metabolic outcomes, utilizing both direct and indirect evidence, and employing NMA to rank the efficacy of various IF forms. Our findings indicate that all IF regimens significantly reduced BW compared to a usual diet, although their effects were less pronounced when compared to CER diet. When considering direct evidence across all metabolic outcomes, IF showed significant improvements in 42% of the comparisons with usual diets and 8% when compared to CER. Whether from direct evidence or NMA, ADF consistently exhibited more favorable outcomes overall. Moreover, ADF emerged as the top-ranked IF approach for enhancing metabolic health, being ranked first in 64.3% of the comparisons and second in 14.3%. These findings suggest that IF may serve as suitable and non-inferior, and, in some cases, superior alternative to improve metabolic health compared to both usual diets and CER.

IF can promote metabolic benefits by shifting metabolism from glucose reliance to using ketone bodies from fatty acids, reducing lipid synthesis and fat storage, thus potentially improving body composition [126]. In addition, the reduction in blood pressure might be attributed to the activation of the parasympathetic nervous system, driven by increased activity of cholinergic neurons in the brainstem [127]. IF can also reduce insulin resistance by lowering body fat, leading to improved insulin sensitivity and glucose metabolism [128]. During fasting, the liver upregulates peroxisome proliferator-activated receptor alpha (PPARα) and peroxisome proliferator-activated receptor-gamma coactivator 1-alpha (PGC-1α), thus enhancing fatty acid oxidation and reducing triglyceride accumulation. This process lowers very low-density lipoprotein cholesterol (VLDL) production and blood LDL-C levels [129].

Previous studies have shown that adherence to CER may decline over time, leading to outcomes that may not align with expectations [13]. Among the meta-analyses we included, two studies investigated adherence to both IF and CER. In the study by Elortegui Pascual et al., adherence percentages ranged from 71.7 to 98% for ADF, 73.5 to 98% for the 5:2 diet, 83 to 89% for TRE, and 80 to 90% for CER [53]. In contrast, Schwingshackl et al. reported an average adherence of 76% (range: 44% to 98%) for 5:2/4:3 diets, while CER had a lower average adherence of 62% (range: 32% to 78%) [37].

In most studies, both IF and CER did not result in significant adverse events, and the probability of side effects was low [15]. Among individuals adhering to a CER diet, 2% to 7% reported mild occurrences of mild nausea, dizziness, feeling cold, constipation, lack of concentration, being preoccupied with food, mood swings or bad temper, mild headache, and decreased energy levels [15]. The implementation of IF diets for most participants resulted in symptoms such as constipation [15, 130], diarrhea [130], dizziness [15, 130], reported hunger [15], mild headaches [15, 25, 130], temporary sleep disturbances [15], and bad breath [15]. Nevertheless, studies have shown that these symptoms tends to improve over time for the majority of participants following an IF eating pattern [15, 25, 130].

We conducted a comprehensive investigation into the effects of IF diets, including TRE, ADF, 5:2/4:3, and STIER diets, on metabolic outcomes including anthropometric measures, blood pressure, glycemic parameters, and lipid profile. Notably, the majority of the best studies we included were published after 2022. Therefore, our umbrella review offers a current and comprehensive synthesis of the evidence on this topic. Furthermore, we undertook a comprehensive comparison between various IF regimens and two reference diets, namely the usual diet and CER. Additionally, we utilized network meta-analysis, which enabled us to evaluate both direct and indirect evidence, to assess the relative efficacy of different IF regimens on metabolic health.

However, it is noteworthy that 63% of the direct evidence available was classified as low or very low quality. Furthermore, the intervention durations varied in studies included in the original meta-analyses, ranging from as short as half a month to as long as 24 months. Therefore, further high-quality research is needed to investigate the effects of different intervention durations and the long-term sustainability of these effects.

## Conclusions

All IF diets demonstrated significant reductions in body weight compared to a usual diet, although their effects were less prominent for other metabolic outcomes and when compared to CER. Among the IF regimens, ADF seemed to yield better results overall across the investigated outcomes, except for HDL-C. However, the majority of direct evidence available had low or very low certainty, underscoring the need for further well-conducted RCTs to strengthen these findings. Nonetheless, considering the available evidence as a whole, IF appears to be a promising dietary strategy that is at least comparable to CER in terms of improving metabolic health.

## Supplementary Information

Additional file 1. Text S1. Details of the study selection process. Table S1. Search strategies. Table S2. List of discrepancies between the initial protocol and the final analysis. Table S3. Criteria and results of the GRADE assessment. Table S4. Criteria and results of the CINeMA assessment. Table S5. Characteristics of original studies within included studies. Table S6. Network evidence of pair-wise comparison with CINeMA. Table S7. Results for meta-regression. Figure S1. Summary of the direct evidence. Figure S2. Risk of bias in the original studies included in the systematic review. Figure S3. Network plots. Figure S4. Results for surface under the cumulative ranking (SUCRA) analaysis. Figure S5. Results for the sensitivity analysis restricted to healthy people with surface under the cumulative ranking (SUCRA).

## Data Availability

No datasets were generated or analysed during the current study.

## Abbreviations

ADF: Alternate-day fasting
AMSTAR2: Assessment of Multiple Systematic Reviews 2
BMI: Body mass index
BW: Body weight
CER: Continuous energy restriction
CIs: Confidence intervals
CINeMA: Confidence in Network Meta-Analysis
DBP: Diastolic blood pressure
FBG: Fasting blood glucose
FFM: Fat-free mass
FI: Fasting insulin
FM: Fat mass
GRADE: Grading of Recommendations, Assessment, Development and Evaluation
HbA1c: Glycosylated hemoglobin
HDL-C: High-density lipoprotein cholesterol
IF: Intermittent fasting
LDL-C: Low-density lipoprotein cholesterol
NMA: Network meta-analysis
PGC-1α: Peroxisome proliferator-activated receptor-gamma coactivator 1-alpha
PPARα: Peroxisome proliferator-activated receptor alpha
RCT: Randomized controlled trial
SBP: Systolic blood pressure
SEs: Standard errors
SUCRA: Surface-under-the-cumulative-ranking-curve analysis
TC: Total cholesterol
TG: Triglycerides
TRE: Time-restricted eating
VLDL: Very low-density lipoprotein cholesterol
WC: Waist circumference
