# Supplementary material for: Effects of different types of intermittent fasting on metabolic outcomes: an umbrella review and network meta-analysis
Source: BMC Med. 2024 Nov 13;22:529. doi: 10.1186/s12916-024-03716-1 (PMC11559166; doi:10.1186/s12916-024-03716-1)
Supplement: Supplementary file 1 — Additional file 1. Text S1. Details of the study selection process. Table S1. Search strategies. Table S2. List of discrepancies between the initial protocol and the final analysis. Table S3. Criteria and results of the GRADE assessment. Table S4. Criteria and results of the CINeMA assessment. Table S5. Characteristics of original studies within included studies. Table S6. Network evidence of pair-wise comparison with CINeMA. Table S7. Results for meta-regression. Figure S1. Summary of the direct evidence. Figure S2. Risk of bias in the original studies included in the systematic review. Figure S3. Network plots. Figure S4. Results for surface under the cumulative ranking (SUCRA) analaysis. Figure S5. Results for the sensitivity analysis restricted to healthy people with surface under the cumulative ranking (SUCRA). [file 12916_2024_3716_MOESM1_ESM.docx]

Content

[Text S1. Details of the study selection process 1](#_Toc175998537)

[Table S1. Search strategies 2](#_Toc175998538)

[Table S2. List of discrepancies between the initial protocol and the final analysis 3](#_Toc175998539)

[Table S3. Criteria and results of the GRADE assessment 4](#_Toc175998540)

[(A) Body weight (BW) 5](#_Toc175998541)

[(B) Body mass index (BMI) 6](#_Toc175998542)

[(C) Fat mass (FM) 7](#_Toc175998543)

[(D) Fat free mass (FFM) 8](#_Toc175998544)

[(E) Waist circumference (WC) 9](#_Toc175998545)

[(F) Systolic blood pressure (SBP) 10](#_Toc175998546)

[(G) Diastolic blood pressure (DBP) 11](#_Toc175998547)

[(H) Fasting blood glucose (FBG) 12](#_Toc175998548)

[(I) Fasting insulin (FI) 13](#_Toc175998549)

[(J) Hemoglobin A1c (HbA1c) 14](#_Toc175998550)

[(K) Triglycerides (TG) 15](#_Toc175998551)

[(L) Total cholesterol (TC) 16](#_Toc175998552)

[(M) Low density lipoprotein cholesterol (LDL-C) 17](#_Toc175998553)

[(N) High density lipoprotein cholesterol (HDL-C) 18](#_Toc175998554)

[Table S4. Criteria and results of the CINeMA assessment 19](#_Toc175998556)

[(A) Body weight (BW) 21](#_Toc175998557)

[(B) Body mass index (BMI) 21](#_Toc175998558)

[(C) Fat mass (FM) 22](#_Toc175998559)

[(D) Fat free mass (FFM) 22](#_Toc175998560)

[(E) Waist circumference (WC) 23](#_Toc175998561)

[(F) Systolic blood pressure (SBP) 23](#_Toc175998562)

[(G) Diastolic blood pressure (DBP) 24](#_Toc175998563)

[(H) Fasting blood glucose (FBG) 24](#_Toc175998564)

[(I) Fasting insulin (FI) 25](#_Toc175998565)

[(J) Hemoglobin A1c (HbA1c) 25](#_Toc175998566)

[(K) Triglycerides (TG) 26](#_Toc175998567)

[(L) Total cholesterol (TC) 26](#_Toc175998568)

[(M) Low density lipoprotein cholesterol (LDL-C) 27](#_Toc175998569)

[(N) High density lipoprotein cholesterol (HDL-C) 27](#_Toc175998570)

[Table S5. Characteristics of original studies within included studies. 28](#_Toc175998555)

[Table S6. Network evidence of pairs-wise comparison with CINeMA. 30](#_Toc175998571)

[(A) Body weight (BW) 30](#_Toc175998572)

[(B) Body mass index (BMI) 30](#_Toc175998573)

[(C) Fat mass (FM) 31](#_Toc175998574)

[(D) Fat free mass/Lean mass (FFM/LM) 31](#_Toc175998575)

[(E) Waist circumference (WC) 32](#_Toc175998576)

[(F) Systolic blood pressure (SBP) 32](#_Toc175998577)

[(G) Diastolic blood pressure (DBP) 33](#_Toc175998578)

[(H) Fasting blood glucose (FBG) 33](#_Toc175998579)

[(I) Fasting insulin (FI) 34](#_Toc175998580)

[(J) Hemoglobin A1c (HbA1c) 34](#_Toc175998581)

[(K) Triglycerides (TG) 35](#_Toc175998582)

[(L) Total cholesterol (TC) 35](#_Toc175998583)

[(M) Low density lipoprotein cholesterol (LDL-C) 36](#_Toc175998584)

[(N) High density lipoprotein cholesterol (HDL-C) 36](#_Toc175998585)

[Table S7. Results for meta-regression 37](#_Toc175998586)

[Figure S1. Summary of the direct evidence 38](#_Toc175998587)

[(A) Body weight (BW) 38](#_Toc175998588)

[(B) Body mass index (BMI) 38](#_Toc175998589)

[(C) Fat mass (FM) 39](#_Toc175998590)

[(D) Fat free mass (FFM) 39](#_Toc175998591)

[(E) Waist circumference (WC) 39](#_Toc175998592)

[(F) Systolic blood pressure (SBP) 40](#_Toc175998593)

[(G) Diastolic blood pressure (DBP) 40](#_Toc175998594)

[(H) Fasting blood glucose (FBG) 40](#_Toc175998595)

[(I) Fasting insulin (FI) 41](#_Toc175998596)

[(J) Hemoglobin A1c (HbA1c) 41](#_Toc175998596)

[(K) Triglycerides (TG) 41](#_Toc175998597)

[(L) Total cholesterol (TC) 42](#_Toc175998598)

[(M) Low density lipoprotein cholesterol (LDL-C) 42](#_Toc175998599)

[(N) High density lipoprotein cholesterol (HDL-C) 42](#_Toc175998600)

[Figure S2. Risk of bias in the original studies included in the systematic review 43](#_Toc175998601)

[Figure S3. Network plots 44](#_Toc175998602)

[(A) Body weight (BW) 44](#_Toc175998603)

[(B) Body mass index (BMI) 44](#_Toc175998604)

[(C) Fat mass (FM) 44](#_Toc175998605)

[(D) Fat free mass/Lean mass (FFM/LM) 45](#_Toc175998606)

[(E) Waist circumference (WC) 45](#_Toc175998607)

[(F) Systolic blood pressure (SBP) 45](#_Toc175998608)

[(G) Diastolic blood pressure (DBP) 46](#_Toc175998609)

[(H) Fasting blood glucose (FBG) 46](#_Toc175998610)

[(I) Fasting insulin (FI) 46](#_Toc175998611)

[(J) Hemoglobin A1c (HbA1c) 47](#_Toc175998612)

[(K) Triglycerides (TG) 47](#_Toc175998613)

[(L) Total cholesterol (TC) 47](#_Toc175998614)

[(M) Low density lipoprotein cholesterol (LDL-C) 48](#_Toc175998615)

[(N) High density lipoprotein cholesterol (HDL-C) 48](#_Toc175998616)

[Figure S4. Results for surface under the cumulative ranking (SUCRA) analaysis 49](#_Toc175998617)

[(A) Body weight (BW) 49](#_Toc175998618)

[(B) Body mass index (BMI) 49](#_Toc175998619)

[(C) Fat mass (FM) 50](#_Toc175998620)

[(D) Fat free mass/Lean mass (FFM/LM) 50](#_Toc175998621)

[(E) Waist circumference (WC) 50](#_Toc175998622)

[(F) Systolic blood pressure (SBP) 51](#_Toc175998623)

[(G) Diastolic blood pressure (DBP) 51](#_Toc175998624)

[(H) Fasting blood glucose (FBG) 51](#_Toc175998625)

[(I) Fasting insulin (FI) 52](#_Toc175998626)

[(J) Hemoglobin A1c (HbA1c) 52](#_Toc175998627)

[(K) Triglycerides (TG) 52](#_Toc175998628)

[(L) Total cholesterol (TC) 53](#_Toc175998629)

[(M) Low density lipoprotein cholesterol (LDL-C) 53](#_Toc175998630)

[(N) High density lipoprotein cholesterol (HDL-C) 54](#_Toc175998631)

[Figure S5. Results for the sensitivity analysis restricted to healthy people with surface under the cumulative ranking (SUCRA) 55](#_Toc175998632)

# Text S1. Details of the study selection process

We included studies that met the following criteria: meta-analyses of randomized controlled trials (RCTs) investigating the association between intermittent fasting (IF) and metabolic health outcomes in humans. We excluded studies that 1) were not meta-analyses, 2) did not investigate IF in relation to metabolic health in humans, or 3) only pooled estimates from observational designs. A total of 39 meta-analyses met the inclusion criteria.

If more than one published meta-analysis on the same comparison was identified, we selected only one meta-analysis for each exposure to avoid including duplicate original studies. To ensure we chose the most representative study, we referred to previous research and applied the following criteria [16,17]:

1. We prioritized the meta-analysis with the largest number of primary studies.

2. If multiple meta-analyses had the same number of studies, we selected the one with the highest quality, as assessed by the Assessment of Multiple Systematic Reviews 2 (AMSTAR2) tool.

3. If the quality ratings were the same, we then chose the meta-analysis with the largest number of participants for the same comparisons.

Ultimately, 10 of the most representative "best studies" were included in our umbrella review. To avoid the duplication of multi-arm primary studies in our network meta-analysis, we extracted data from the primary studies within these best studies, totaling 40 studies, for the study-level network meta-analysis.

# Table S1. Search strategies

| Database | Search terms |
| --- | --- |
| PubMed | ("intermittent feeding" OR "intermittent eating" OR "intermittent diet" OR "intermittent meal" OR "intermittent fasting" OR "intermittent calorie restriction" OR "intermittent caloric restriction" OR "intermittent energy restriction" OR "time-restricted feeding" OR "time restricted feeding" OR "time-restricted eating" OR "time restricted eating" OR "time-restricted diet" OR "time restricted diet" OR "time-restricted meal" OR "time restricted meal" OR "time-restricted fasting" OR "time restricted fasting" OR "alternate-day feeding" OR "alternate day feeding" OR "alternate-day eating" OR "alternate day eating" OR "alternate-day diet" OR "alternate day diet" OR "alternate-day meal" OR "alternate day meal" OR "alternate-day fasting" OR "alternate day fasting" OR "5:2 diet") AND ("systematic review" OR "systematic literature review" OR "meta-analysis" OR "meta analysis" OR "meta-analyses" OR "meta analyses" OR "Systematic Review"[Publication Type] OR "Meta-Analysis"[Publication Type]) |
| EMBASE | ('intermittent feeding' OR 'intermittent eating' OR 'intermittent diet' OR 'intermittent meal' OR 'intermittent fasting' OR 'intermittent calorie restriction' OR 'intermittent caloric restriction' OR 'intermittent energy restriction' OR 'time-restricted feeding' OR 'time restricted feeding' OR 'time-restricted eating' OR 'time restricted eating' OR 'time-restricted diet' OR 'time restricted diet' OR 'time-restricted meal' OR 'time restricted meal' OR 'time-restricted fasting' OR 'time restricted fasting' OR 'alternate-day feeding' OR 'alternate day feeding' OR 'alternate-day eating' OR 'alternate day eating' OR 'alternate-day diet' OR 'alternate day diet' OR 'alternate-day meal' OR 'alternate day meal' OR 'alternate-day fasting' OR 'alternate day fasting' OR '5:2 diet') AND ('systematic review' OR 'systematic literature review' OR 'meta-analysis' OR 'meta analysis' OR 'meta-analyses' OR 'meta analyses' OR 'systematic review':it OR 'meta-analysis':it) |
| Cochrane | ("intermittent feeding" OR "intermittent eating" OR "intermittent diet" OR "intermittent meal" OR "intermittent fasting" OR "intermittent calorie restriction" OR "intermittent caloric restriction" OR "intermittent energy restriction" OR "time-restricted feeding" OR "time restricted feeding" OR "time-restricted eating" OR "time restricted eating" OR "time-restricted diet" OR "time restricted diet" OR "time-restricted meal" OR "time restricted meal" OR "time-restricted fasting" OR "time restricted fasting" OR "alternate-day feeding" OR "alternate day feeding" OR "alternate-day eating" OR "alternate day eating" OR "alternate-day diet" OR "alternate day diet" OR "alternate-day meal" OR "alternate day meal" OR "alternate-day fasting" OR "alternate day fasting" OR "5:2 diet") AND ("systematic review" OR "systematic literature review" OR meta-analysis OR "meta analysis" OR meta-analyses OR "meta analyses" OR "Systematic Review":pt OR Meta-Analysis:pt) |

# Table S2. List of discrepancies between the initial protocol and the final analysis

| Initial protocol | Differences | Explanation |
| --- | --- | --- |
| We included systematic review studies that did not conduct meta-analysis for our analysis. | We excluded systematic review studies that did not include meta-analysis in our analysis. | Since systematic reviews did not incorporate quantitative analysis (meta-analysis), it was challenging to determine the precise impact of different types of intermittent fasting on metabolic health outcomes. Therefore, conducting a network meta-analysis was not feasible. |

# Table S3. Criteria and results of the GRADE assessment

**Criteria** **for each domain**

1. Risk of bias: If included studies with weights over 50% were considered to have a high risk of bias in at least one domain, we would evaluate the risk of bias downward [37, 58-59].

2. Inconsistency: a. If the *I*² of meta-analysis 40~75%, we would classify inconsistency as serious [60].

b. If the *I*² of meta-analysis ≥ 75%, we would classify inconsistency as very serious [60].

c. If only one study was included, we did not make any downgrading, but we make a note

3. Indirectness: If there was indirectness in the study regarding the population, intervention, or outcome measures, we would downgrade [61].

4. Imprecision: If the sample size was small (total population <400), we would downgrade [62].

5. Publication bias: If visual inspection demonstrates an asymmetrical funnel plot or statistical tests for asymmetry was positive, we would downgrade [63].

6. When a meta-analysis includes only a small number of studies, its evidence certainty is often downgraded due to the risk of publication bias and imprecision [62-64]. However, if only one original study is included, publication bias cannot be assessed, and inconsistency cannot be evaluated. To avoid overestimating the certainty, we adopted a cautious approach to rate such evidence as having very low certainty.

## (A) Body weight (BW)

| **Certainty assessment** | | | | | | | **Effect** | **Certainty** |
| --- | --- | --- | --- | --- | --- | --- | --- | --- |
| **№ of studies** | **Study design** | **Risk of bias** | **Inconsistency** | **Indirectness** | **Imprecision** | **Publication bias** | **Relative** |  |
|  |  |  |  |  |  |  | **(95% CI)** |  |
| **[TRE] - [Usual]** [21] | | | | | | | | |
| 5 | randomised trials | serious^a^ | not serious | not serious | serious^b^ | none | kg -0.38 | ⨁⨁◯◯ |
|  |  |  |  |  |  |  | (-0.71 to -0.05) | Low |
| **[TRE] - [Usual/CER]** [50] | | | | | | | | |
| 17 | randomised trials | not serious | very serious^c^ | not serious | not serious | none | kg -1.60 | ⨁⨁◯◯ |
|  |  |  |  |  |  |  | (-2.27 to -0.93) | Low |
| **[TRE] - [CER]** [55] | | | | | | | | |
| 8 | randomised trials | not serious | serious^d^ | not serious | not serious | none | kg -2.41 | ⨁⨁⨁◯ |
|  |  |  |  |  |  |  | (-5.14 to 0.33) | Moderate |
| **[TRE] - [ADF]** [26] | | | | | | | | |
| 1 | randomised trials | not serious | not serious^e^ | not serious | serious^b^ | none^e^ | kg 0.39 | ⨁◯◯◯ |
|  |  |  |  |  |  |  | (-1.68 to 2.46) | Very low |
| **[ADF] - [Usual]** [25] | | | | | | | | |
| 7 | randomised trials | not serious | very serious^c^ | not serious | serious^b^ | none | kg -4.30 | ⨁◯◯◯ |
|  |  |  |  |  |  |  | (-5.54 to -3.05) | Very low |
| **[ADF] - [CER]** [53] | | | | | | | | |
| 8 | randomised trials | not serious | very serious^c^ | not serious | serious^b^ | none | kg -0.17 | ⨁◯◯◯ |
|  |  |  |  |  |  |  | (-1.03 to 0.69) | Very low |
| **[5:2] - [CER]** [54] | | | | | | | | |
| 8 | randomised trials | not serious | not serious | not serious | not serious | none^f^ | kg -0.02 | ⨁⨁⨁⨁ |
|  |  |  |  |  |  |  | (-0.17 to 0.13) | High |
| **[STIER] - [CER]** [33] | | | | | | | | |
| 5 | randomised trials | serious^a^ | not serious | not serious | serious^b^ | none | kg -1.36 | ⨁⨁◯◯ |
|  |  |  |  |  |  |  | (-3.23 to 0.51) | Low |
| **[5:2/4:3] - [Usual]** [37] | | | | | | | | |
| 6 | randomised trials | serious^a^ | not serious | not serious | serious^b^ | none | kg -4.83 | ⨁⨁◯◯ |
|  |  |  |  |  |  |  | (-5.46 to -4.21) | Low |
| **[5:2/4:3] - [CER]** [37] | | | | | | | | |
| 13 | randomised trials | serious^a^ | not serious | not serious | not serious | none | kg -0.55 | ⨁⨁⨁◯ |
|  |  |  |  |  |  |  | (-1.01 to -0.09) | Moderate |

**Explanations**

a. The included studies with weights over 50% were considered to have a high risk of bias in at least one domain.

b. small sample size (total population <400)

c. I-square ≥ 75%

d. I-square 40~75%

e. only one study

f. no analysis

## (B) Body mass index (BMI)

| **Certainty assessment** | | | | | | | **Effect** | **Certainty** |
| --- | --- | --- | --- | --- | --- | --- | --- | --- |
| **№ of studies** | **Study design** | **Risk of bias** | **Inconsistency** | **Indirectness** | **Imprecision** | **Publication bias** | **Relative** |  |
|  |  |  |  |  |  |  | **(95% CI)** |  |
| **[TRE] - [CER/Usual]** [50] | | | | | | | | |
| 8 | randomised trials | not serious | very serious^a^ | not serious | not serious | none | kg/m² -0.53 | ⨁⨁◯◯ |
|  |  |  |  |  |  |  | (-1.10 to 0.04) | Low |
| **[TRE] - [CER]** [55] | | | | | | | | |
| 4 | randomised trials | not serious | serious^d^ | not serious | not serious | none | kg/m² -0.66 | ⨁⨁⨁◯ |
|  |  |  |  |  |  |  | (-2.22 to 0.91) | Moderate |
| **[TRE] - [ADF]** [26] | | | | | | | | |
| 1 | randomised trials | not serious | not serious^b^ | not serious | serious^c^ | none^b^ | kg/m² 0.45 | ⨁◯◯◯ |
|  |  |  |  |  |  |  | (0.01 to 0.89) | Very low |
| **[ADF] - [Usual]** [26] | | | | | | | | |
| 4 | randomised trials | not serious | serious^d^ | not serious | serious^c^ | none | kg/m² -0.96 | ⨁⨁◯◯ |
|  |  |  |  |  |  |  | (-1.42 to -0.50) | Low |
| **[ADF] - [CER]** [55] | | | | | | | | |
| 5 | randomised trials | not serious | serious^d^ | not serious | not serious | none | kg/m² -0.66 | ⨁⨁⨁◯ |
|  |  |  |  |  |  |  | (-1.57 to 0.24) | Moderate |
| **[5:2] - [CER]** [[54](#_ENREF_54)] | | | | | | | | |
| 5 | randomised trials | not serious | not serious | not serious | not serious | none^e^ | kg/m² 0.08 | ⨁⨁⨁⨁ |
|  |  |  |  |  |  |  | (-0.11 to 0.27) | High |

**Explanations**

a. I-square ≥ 75%

b. only one study

c. small sample size (total population <400)

d. I-square 40~75%

e. no analysis

## (C) Fat mass (FM)

| **Certainty assessment** | | | | | | | **Effect** | **Certainty** |
| --- | --- | --- | --- | --- | --- | --- | --- | --- |
| **№ of studies** | **Study design** | **Risk of bias** | **Inconsistency** | **Indirectness** | **Imprecision** | **Publication bias** | **Relative** |  |
|  |  |  |  |  |  |  | **(95% CI)** |  |
| **[TRE] - [Usual/CER]** [50] | | | | | | | | |
| 13 | randomised trials | not serious | not serious | not serious | not serious | none | kg -1.48 | ⨁⨁⨁⨁ |
|  |  |  |  |  |  |  | (-1.59 to -1.38) | High |
| **[TRE] - [CER]** [55] | | | | | | | | |
| 5 | randomised trials | not serious | serious^a^ | not serious | not serious | none | kg -2.58 | ⨁⨁⨁◯ |
|  |  |  |  |  |  |  | (-4.55 to 0.61) | Moderate |
| **[TRE] - [ADF]** [26] | | | | | | | | |
| 1 | randomised trials | not serious | not serious^b^ | not serious | serious^c^ | none^b^ | kg 0.55 | ⨁◯◯◯ |
|  |  |  |  |  |  |  | (-0.30 to 1.40) | Very low |
| **[ADF] - [Usual]** [[25](#_ENREF_25)] | | | | | | | | |
| 6 | randomised trials | serious^d^ | very serious^e^ | not serious | serious^c^ | none | kg -4.96 | ⨁◯◯◯ |
|  |  |  |  |  |  |  | (-8.08 to 1.85) | Very low |
| **[ADF] - [CER]** [55] | | | | | | | | |
| 6 | randomised trials | not serious | very serious^e^ | not serious | not serious | none | kg -2.80 | ⨁⨁◯◯ |
|  |  |  |  |  |  |  | (-6.34 to 0.74) | Low |
| **[5:2/4:3] - [Usual]** [[37](#_ENREF_37)] | | | | | | | | |
| 6 | randomised trials | not serious | serious^a^ | not serious | serious^c^ | none | kg -2.54 | ⨁⨁◯◯ |
|  |  |  |  |  |  |  | (-3.78 to -1.31) | Low |
| **[5:2/4:3] - [CER]** [[37](#_ENREF_37)] | | | | | | | | |
| 10 | randomised trials | not serious | not serious | not serious | not serious | none | kg -0.66 | ⨁⨁⨁⨁ |
|  |  |  |  |  |  |  | (-1.14 to -0.19) | High |
| **[5:2] - [CER]** [[56](#_ENREF_56)] | | | | | | | | |
| 5 | randomised trials | not serious | not serious | not serious | not serious | none^f^ | kg 0.06 | ⨁⨁⨁⨁ |
|  |  |  |  |  |  |  | (-1.02 to 1.14) | High |
| **[TRE] - [Usual]** [[21](#_ENREF_21)] | | | | | | | | |
| 4 | randomised trials | serious^d^ | not serious | not serious | serious^c^ | none | kg -0.83 | ⨁⨁◯◯ |
|  |  |  |  |  |  |  | (-1.89 to 0.24) | Low |

**Explanations**

a. I-square 40~75%

b. only one study

c. small sample size (total population <400)

d. The included studies with weights over 50% were considered to have a high risk of bias in at least one domain.

e. I-square ≥ 75%

f. no analysis

## (D) Fat free mass (FFM)

| **Certainty assessment** | | | | | | | **Effect** | **Certainty** |
| --- | --- | --- | --- | --- | --- | --- | --- | --- |
| **№ of studies** | **Study design** | **Risk of bias** | **Inconsistency** | **Indirectness** | **Imprecision** | **Publication bias** | **Relative** |  |
|  |  |  |  |  |  |  | **(95% CI)** |  |
| **[TRE] - [Usual]** [[21](#_ENREF_21)] | | | | | | | | |
| 4 | randomised trials | serious^a^ | not serious | not serious | serious^b^ | none | kg 0.00 | ⨁⨁◯◯ |
|  |  |  |  |  |  |  | (-0.78 to 0.79) | Low |
| **[TRE] - [CER]** [55] | | | | | | | | |
| 5 | randomised trials | not serious | not serious | not serious | not serious | none | kg -1.27 | ⨁⨁⨁⨁ |
|  |  |  |  |  |  |  | (-2.82 to 0.29) | High |
| **[ADF] - [CER]** [[54](#_ENREF_54)] | | | | | | | | |
| 5 | randomised trials | not serious | not serious | not serious | serious^b^ | none^c^ | kg 0.08 | ⨁⨁⨁◯ |
|  |  |  |  |  |  |  | (-0.13 to 0.30) | Moderate |
| **[5:2] - [CER]** [[56](#_ENREF_56)] | | | | | | | | |
| 5 | randomised trials | not serious | not serious | not serious | not serious | none^c^ | kg -0.23 | ⨁⨁⨁⨁ |
|  |  |  |  |  |  |  | (-0.97 to 0.51) | High |

**Explanations**

a. The included studies with weights over 50% were considered to have a high risk of bias in at least one domain.

b. small sample size (total population <400)

c. no analysis

## (E) Waist circumference (WC)

| **Certainty assessment** | | | | | | | **Effect** | **Certainty** |
| --- | --- | --- | --- | --- | --- | --- | --- | --- |
| **№ of studies** | **Study design** | **Risk of bias** | **Inconsistency** | **Indirectness** | **Imprecision** | **Publication bias** | **Relative** |  |
|  |  |  |  |  |  |  | **(95% CI)** |  |
| **[TRE] - [Usual/CER]** [50] | | | | | | | | |
| 7 | randomised trials | not serious | not serious | not serious | not serious | none | cm -0.07 | ⨁⨁⨁⨁ |
|  |  |  |  |  |  |  | (-0.98 to 0.83) | High |
| **[TRE] - [CER]** [55] | | | | | | | | |
| 6 | randomised trials | not serious | not serious | not serious | not serious | none | cm -3.66 | ⨁⨁⨁⨁ |
|  |  |  |  |  |  |  | (-6.08 to 1.24) | High |
| **[TRE] - [ADF]** [26] | | | | | | | | |
| 1 | randomised trials | not serious | not serious^b^ | not serious | serious^c^ | none^b^ | cm 0.39 | ⨁◯◯◯ |
|  |  |  |  |  |  |  | (-0.95 to 1.73) | Very low |
| **[ADF] - [Usual]** [26] | | | | | | | | |
| 2 | randomised trials | not serious | serious^a^ | not serious | serious^c^ | none | cm -2.41 | ⨁⨁◯◯ |
|  |  |  |  |  |  |  | (-4.95 to 0.13) | Low |
| **[ADF] - [CER]** [55] | | | | | | | | |
| 3 | randomised trials | not serious | not serious | not serious | not serious | none | cm -1.37 | ⨁⨁⨁⨁ |
|  |  |  |  |  |  |  | (-4.08 to 1.34) | High |
| **[5:2] - [CER]** [[54](#_ENREF_54)] | | | | | | | | |
| 5 | randomised trials | not serious | not serious | not serious | serious^c^ | none^e^ | cm -0.06 | ⨁⨁⨁◯ |
|  |  |  |  |  |  |  | (-0.26 to 0.14) | Moderate |
| **[5:2/4:3] - [CER]** [[37](#_ENREF_37)] | | | | | | | | |
| 8 | randomised trials | not serious | not serious | not serious | not serious | none | cm -0.57 | ⨁⨁⨁⨁ |
|  |  |  |  |  |  |  | (-1.56 to 0.41) | High |
| **[5:2/4:3] - [Usual]** [[37](#_ENREF_37)] | | | | | | | | |
| 2 | randomised trials | not serious | not serious | not serious | serious^c^ | none | cm -1.73 | ⨁⨁⨁◯ |
|  |  |  |  |  |  |  | (-3.69 to 0.24) | Moderate |

**Explanations**

a. I-square 40~75%

b. only one study

c. small sample size (total population <400)

d. I-square ≥ 75%

e. no analysis

## (F) Systolic blood pressure (SBP)

| **Certainty assessment** | | | | | | | **Effect** | **Certainty** |
| --- | --- | --- | --- | --- | --- | --- | --- | --- |
| **№ of studies** | **Study design** | **Risk of bias** | **Inconsistency** | **Indirectness** | **Imprecision** | **Publication bias** | **Relative** |  |
|  |  |  |  |  |  |  | **(95% CI)** |  |
| **[TRE] - [Usual/CER]** [50] | | | | | | | | |
| 8 | randomised trials | not serious | very serious^a^ | not serious | not serious | none | mmHg -2.46 | ⨁⨁◯◯ |
|  |  |  |  |  |  |  | (-6.43 to 1.51) | Low |
| **[TRE] - [CER]** [55] | | | | | | | | |
| 4 | randomised trials | not serious | not serious | not serious | not serious | none | mmHg -3.19 | ⨁⨁⨁⨁ |
|  |  |  |  |  |  |  | (-7.63 to 1.26) | High |
| **[ADF] - [Usual]** [[25](#_ENREF_25)] | | | | | | | | |
| 4 | randomised trials | serious^b^ | very serious^a^ | not serious | serious^c^ | none | mmHg -4.42 | ⨁◯◯◯ |
|  |  |  |  |  |  |  | (-7.35 to -1.49) | Very low |
| **[ADF] - [CER]** [55] | | | | | | | | |
| 3 | randomised trials | not serious | very serious^a^ | not serious | not serious | none | mmHg -4.34 | ⨁⨁◯◯ |
|  |  |  |  |  |  |  | (-14.50 to 5.83) | Low |
| **[5:2] - [CER]** [[54](#_ENREF_54)] | | | | | | | | |
| 4 | randomised trials | not serious | not serious | not serious | serious^c^ | none^d^ | mmHg 0.20 | ⨁⨁⨁◯ |
|  |  |  |  |  |  |  | (-0.03 to 0.42) | Moderate |
| **[5:2/4:3] - [Usual]** [[37](#_ENREF_37)] | | | | | | | | |
| 5 | randomised trials | not serious | not serious | not serious | serious^c^ | none | mmHg -6.11 | ⨁⨁⨁◯ |
|  |  |  |  |  |  |  | (-9.59 to -2.64) | Moderate |
| **[5:2/4:3] - [CER]** [[37](#_ENREF_37)] | | | | | | | | |
| 8 | randomised trials | not serious | not serious | not serious | not serious | none | mmHg 0.24 | ⨁⨁⨁⨁ |
|  |  |  |  |  |  |  | (-2.08 to 2.55) | High |
| **[TRE] - [Usual]** [[21](#_ENREF_21)] | | | | | | | | |
| 2 | randomised trials | serious^b^ | very serious^a^ | not serious | serious^c^ | none | mmHg -2.27 | ⨁◯◯◯ |
|  |  |  |  |  |  |  | (-19.52 to 14.98) | Very low |

**Explanations**

a. I-square ≥ 75%

b. The included studies with weights over 50% were considered to have a high risk of bias in at least one domain.

c. small sample size (total population <400)

d. no analysis

## (G) Diastolic blood pressure (DBP)

| **Certainty assessment** | | | | | | | **Effect** | **Certainty** |
| --- | --- | --- | --- | --- | --- | --- | --- | --- |
| **№ of studies** | **Study design** | **Risk of bias** | **Inconsistency** | **Indirectness** | **Imprecision** | **Publication bias** | **Relative** |  |
|  |  |  |  |  |  |  | **(95% CI)** |  |
| **[TRE] - [Usual/CER]** [50] | | | | | | | | |
| 8 | randomised trials | not serious | very serious^a^ | not serious | not serious | none | mmHg -1.78 | ⨁⨁◯◯ |
|  |  |  |  |  |  |  | (-4.72 to 1.16) | Low |
| **[TRE] - [CER]** [55] | | | | | | | | |
| 4 | randomised trials | not serious | not serious | not serious | serious^b^ | none | mmHg -3.47 | ⨁⨁⨁◯ |
|  |  |  |  |  |  |  | (-7.00 to 0.06) | Moderate |
| **[ADF] - [Usual]** [[25](#_ENREF_25)] | | | | | | | | |
| 4 | randomised trials | serious^c^ | very serious^a^ | not serious | serious^b^ | none | mmHg -3.41 | ⨁◯◯◯ |
|  |  |  |  |  |  |  | (-5.91 to -0.92) | Very low |
| **[ADF] - [CER]** [[54](#_ENREF_54)] | | | | | | | | |
| 3 | randomised trials | not serious | not serious | not serious | serious^b^ | none^d^ | mmHg -0.04 | ⨁⨁⨁◯ |
|  |  |  |  |  |  |  | (-0.29 to 0.22) | Moderate |
| **[5:2] - [CER]** [[54](#_ENREF_54)] | | | | | | | | |
| 3 | randomised trials | not serious | not serious | not serious | serious^b^ | none^d^ | mmHg -0.04 | ⨁⨁⨁◯ |
|  |  |  |  |  |  |  | (-0.30 to 0.22) | Moderate |
| **[TRE] - [Usual]** [[21](#_ENREF_21)] | | | | | | | | |
| 2 | randomised trials | serious^c^ | very serious^a^ | not serious | serious^b^ | none | mmHg -2.76 | ⨁◯◯◯ |
|  |  |  |  |  |  |  | (-16.27 to 10.75) | Very low |

**Explanations**

a. I-square ≥ 75%

b. small sample size (total population <400)

c. The included studies with weights over 50% were considered to have a high risk of bias in at least one domain.

d. no analysis

## (H) Fasting blood glucose (FBG)

| **Certainty assessment** | | | | | | | **Effect** | **Certainty** |
| --- | --- | --- | --- | --- | --- | --- | --- | --- |
| **№ of studies** | **Study design** | **Risk of bias** | **Inconsistency** | **Indirectness** | **Imprecision** | **Publication bias** | **Relative** |  |
|  |  |  |  |  |  |  | **(95% CI)** |  |
| **[TRE] - [Usual/CER]** [50] | | | | | | | | |
| 12 | randomised trials | not serious | very serious^a^ | not serious | not serious | none | mg/dL -4.08 | ⨁⨁◯◯ |
|  |  |  |  |  |  |  | (-7.74 to -0.42) | Low |
| **[TRE] - [ADF]** [26] | | | | | | | | |
| 1 | randomised trials | not serious | not serious^b^ | not serious | serious^c^ | none^b^ | mg/dL -2.52 | ⨁◯◯◯ |
|  |  |  |  |  |  |  | (-8.01 to 2.97) | Very low |
| **[ADF] - [Usual]** [[25](#_ENREF_25)] | | | | | | | | |
| 4 | randomised trials | not serious | very serious^a^ | not serious | serious^c^ | none | mg/dL -3.02 | ⨁◯◯◯ |
|  |  |  |  |  |  |  | (-6.52 to 0.48) | Very low |
| **[ADF] - [CER]** [26] | | | | | | | | |
| 3 | randomised trials | not serious | serious^f^ | not serious | serious^c^ | none | mg/dL -1.34 | ⨁⨁◯◯ |
|  |  |  |  |  |  |  | (-4.74 to 2.06) | Low |
| **[5:2] - [CER]** [[54](#_ENREF_54)] | | | | | | | | |
| 7 | randomised trials | not serious | very serious^a^ | not serious | not serious | none^d^ | mg/dL 0.15 | ⨁⨁◯◯ |
|  |  |  |  |  |  |  | (-0.02 to 0.33) | Low |
| **[TRE] - [CER]** [[54](#_ENREF_54)] | | | | | | | | |
| 1 | randomised trials | very serious^e^ | not serious^b^ | not serious | serious^c^ | none^b^ | mg/dL -0.09 | ⨁◯◯◯ |
|  |  |  |  |  |  |  | (-7.74 to -0.42) | Very low |
| **[5:2/4:3] - [Usual]** [[37](#_ENREF_37)] | | | | | | | | |
| 4 | randomised trials | not serious | serious^f^ | not serious | serious^c^ | none | mg/dL -2.16 | ⨁⨁◯◯ |
|  |  |  |  |  |  |  | (-7.38 to 3.24) | Low |
| **[5:2/4:3] - [CER]** [[37](#_ENREF_37)] | | | | | | | | |
| 8 | randomised trials | not serious | not serious | not serious | not serious | none | mg/dL 1.08 | ⨁⨁⨁⨁ |
|  |  |  |  |  |  |  | (-0.54 to 2.70) | High |
| **[TRE] - [Usual]** [[21](#_ENREF_21)] | | | | | | | | |
| 4 | randomised trials | serious^g^ | not serious | not serious | serious^c^ | none | mg/dL -2.45 | ⨁⨁◯◯ |
|  |  |  |  |  |  |  | (-4.72 to -0.17) | Low |

**Explanations**

a. I-square ≥ 75%

b. only one study

c. small sample size (total population <400)

d. no analysis

e. Only one study was included, and the risk of bias in this study was assessed as high risk.

f. I-square 40~75%

g. The included studies with weights over 50% were considered to have a high risk of bias in at least one domain.

## (I) Fasting insulin (FI)

| **Certainty assessment** | | | | | | | **Effect** | **Certainty** |
| --- | --- | --- | --- | --- | --- | --- | --- | --- |
| **№ of studies** | **Study design** | **Risk of bias** | **Inconsistency** | **Indirectness** | **Imprecision** | **Publication bias** | **Relative** |  |
|  |  |  |  |  |  |  | **(95% CI)** |  |
| **[ADF] - [Usual]** [26] | | | | | | | | |
| 2 | randomised trials | not serious | not serious | not serious | serious^a^ | none | μU/mL -1.50 | ⨁⨁⨁◯ |
|  |  |  |  |  |  |  | (-10.11 to 7.11) | Moderate |
| **[ADF] - [CER]** [26] | | | | | | | | |
| 3 | randomised trials | not serious | serious^g^ | not serious | serious^a^ | none | μU/mL 0.12 | ⨁⨁◯◯ |
|  |  |  |  |  |  |  | (-2.34 to 2.59) | Low |
| **[TRE] - [CER]** [[54](#_ENREF_54)] | | | | | | | | |
| 1 | randomised trials | very serious^c^ | not serious^d^ | not serious | serious^a^ | none^d^ | μU/mL 0.02 | ⨁◯◯◯ |
|  |  |  |  |  |  |  | (-0.46 to 0.51) | Very low |
| **[5:2] - [CER]** [[54](#_ENREF_54)] | | | | | | | | |
| 4 | randomised trials | not serious | very serious^b^ | not serious | serious^a^ | none^e^ | μU/mL -0.09 | ⨁◯◯◯ |
|  |  |  |  |  |  |  | (-0.31 to 0.14) | Very low |
| **[TRE] - [Usual]** [[21](#_ENREF_21)] | | | | | | | | |
| 3 | randomised trials | serious^f^ | serious^g^ | not serious | serious^a^ | none | μU/mL -0.69 | ⨁◯◯◯ |
|  |  |  |  |  |  |  | (-1.64 to 0.26) | Very low |

**Explanations**

a. small sample size (total population <400)

b. I-square ≥ 75%

c. Only one study was included, and the risk of bias in this study was assessed as high risk.

d. only one study

e. no analysis

f. The included studies with weights over 50% were considered to have a high risk of bias in at least one domain.

g. I-square 40~75%

## (J) Hemoglobin A1c (HbA1c)

| **Certainty assessment** | | | | | | | **Effect** | **Certainty** |
| --- | --- | --- | --- | --- | --- | --- | --- | --- |
| **№ of studies** | **Study design** | **Risk of bias** | **Inconsistency** | **Indirectness** | **Imprecision** | **Publication bias** | **Relative** |  |
|  |  |  |  |  |  |  | **(95% CI)** |  |
| **[TRE] - [Usual/CER]** [50] | | | | | | | | |
| 6 | randomised trials | not serious | very serious^a^ | not serious | serious^b^ | none | -0.11% | ⨁◯◯◯ |
|  |  |  |  |  |  |  | (-0.50 to 0.27) | Very low |
| **[TRE] - [CER]** [[54](#_ENREF_54)] | | | | | | | | |
| 1 | randomised trials | very serious^c^ | not serious^d^ | not serious | serious^b^ | none^e^ | -0.01% | ⨁◯◯◯ |
|  |  |  |  |  |  |  | (-0.50 to 0.47) | Very low |
| **[5:2] - [CER]** [[54](#_ENREF_54)] | | | | | | | | |
| 4 | randomised trials | not serious | serious^f^ | not serious | not serious^b^ | none^e^ | 0.01% | ⨁⨁⨁◯ |
|  |  |  |  |  |  |  | (-0.19 to 0.21) | Moderate |
| **[5:2/4:3] - [Usual]** [[37](#_ENREF_37)] | | | | | | | | |
| 1 | randomised trials | not serious | not serious^d^ | not serious | serious^b^ | none^e^ | 0% | ⨁◯◯◯ |
|  |  |  |  |  |  |  | (-0.08 to 0.08) | Very low |
| **[5:2/4:3] - [CER]** [[37](#_ENREF_37)] | | | | | | | | |
| 6 | randomised trials | not serious | not serious | not serious | not serious | none | -0.01% | ⨁⨁⨁⨁ |
|  |  |  |  |  |  |  | (-0.07 to 0.06) | High |

**Explanations**

a. I-square ≥ 75%

b. small sample size (total population <400)

c. Only one study was included, and the risk of bias in this study was assessed as high risk.

d. only one study

e. no analysis

f. I-square 40~75%

## (K) Triglycerides (TG)

| **Certainty assessment** | | | | | | | **Effect** | **Certainty** |
| --- | --- | --- | --- | --- | --- | --- | --- | --- |
| **№ of studies** | **Study design** | **Risk of bias** | **Inconsistency** | **Indirectness** | **Imprecision** | **Publication bias** | **Relative** |  |
|  |  |  |  |  |  |  | **(95% CI)** |  |
| **[TRE] - [Usual/CER]** [50] | | | | | | | | |
| 13 | randomised trials | not serious | very serious^a^ | not serious | not serious | none | mg/dL -8.64 | ⨁⨁◯◯ |
|  |  |  |  |  |  |  | (-18.01 to 0.73) | Low |
| **[TRE] - [ADF]** [26] | | | | | | | | |
| 1 | randomised trials | not serious | not serious^b^ | not serious | serious^c^ | none^b^ | mg/dL 16.81 | ⨁◯◯◯ |
|  |  |  |  |  |  |  | (-29.84 to 63.47) | Very low |
| **[ADF] - [Usual]** [[25](#_ENREF_25)] | | | | | | | | |
| 5 | randomised trials | not serious | very serious^a^ | not serious | serious^c^ | none | mg/dL -11.27 | ⨁◯◯◯ |
|  |  |  |  |  |  |  | (-20.53 to -2.00) | Very low |
| **[ADF] - [CER]** [26] | | | | | | | | |
| 3 | randomised trials | not serious | serious^e^ | not serious | serious^c^ | none | mg/dL -5.87 | ⨁⨁◯◯ |
|  |  |  |  |  |  |  | (-32.06 to 20.32) | Low |
| **[TRE] - [CER]** [[54](#_ENREF_54)] | | | | | | | | |
| 1 | randomised trials | very serious^d^ | not serious^b^ | not serious | serious^c^ | none^b^ | mg/dL -0.02 | ⨁◯◯◯ |
|  |  |  |  |  |  |  | (-0.51 to 0.47) | Very low |
| **[5:2] - [CER]** [[54](#_ENREF_54)] | | | | | | | | |
| 6 | randomised trials | not serious | not serious | not serious | not serious | none^f^ | mg/dL 0.06 | ⨁⨁⨁⨁ |
|  |  |  |  |  |  |  | (-0.12 to 0.24) | High |
| **[5:2/4:3] - [Usual]** [[37](#_ENREF_37)] | | | | | | | | |
| 5 | randomised trials | not serious | serious^e^ | not serious | serious^c^ | none | mg/dL -17.71 | ⨁⨁◯◯ |
|  |  |  |  |  |  |  | (-33.66 to -2.66) | Low |
| **[5:2/4:3] - [CER]** [[37](#_ENREF_37)] | | | | | | | | |
| 8 | randomised trials | not serious | not serious | not serious | not serious | none | mg/dL -3.54 | ⨁⨁⨁⨁ |
|  |  |  |  |  |  |  | (-11.51 to 5.31) | High |
| **[TRE] - [Usual]** [[21](#_ENREF_21)] | | | | | | | | |
| 4 | randomised trials | serious^g^ | very serious^a^ | not serious | serious^c^ | none | mg/dL 6.24 | ⨁◯◯◯ |
|  |  |  |  |  |  |  | (-13.40 to 25.88) | Very low |

**Explanations**

a. I-square ≥ 75%

b. only one study

c. small sample size (total population <400)

d. Only one study was included, and the risk of bias in this study was assessed as high risk.

e. I-square 40~75%

f. no analysis

g. The included studies with weights over 50% were considered to have a high risk of bias in at least one domain.

## (L) Total cholesterol (TC)

| **Certainty assessment** | | | | | | | **Effect** | **Certainty** |
| --- | --- | --- | --- | --- | --- | --- | --- | --- |
| **№ of studies** | **Study design** | **Risk of bias** | **Inconsistency** | **Indirectness** | **Imprecision** | **Publication bias** | **Relative** |  |
|  |  |  |  |  |  |  | **(95% CI)** |  |
| **[TRE] - [Usual/CER]** [50] | | | | | | | | |
| 10 | randomised trials | not serious | not serious | not serious | not serious | none | mg/dL -6.10 | ⨁⨁⨁⨁ |
|  |  |  |  |  |  |  | (-7.86 to -4.34) | High |
| **[TRE] - [ADF]** [26] | | | | | | | | |
| 1 | randomised trials | not serious | not serious^a^ | not serious | serious^b^ | none^a^ | mg/dL 8.49 | ⨁◯◯◯ |
|  |  |  |  |  |  |  | (-6.09 to 23.08) | Very low |
| **[ADF] - [Usual]** [[25](#_ENREF_25)] | | | | | | | | |
| 5 | randomised trials | not serious | very serious^c^ | not serious | serious^b^ | none | mg/dL -11.32 | ⨁◯◯◯ |
|  |  |  |  |  |  |  | (-18.20 to -4.44) | Very low |
| **[ADF] - [CER]** [26] | | | | | | | | |
| 3 | randomised trials | not serious | not serious | not serious | serious^b^ | none | mg/dL -2.79 | ⨁⨁⨁◯ |
|  |  |  |  |  |  |  | (-11.31 to 5.74) | Moderate |
| **[TRE] - [CER]** [[54](#_ENREF_54)] | | | | | | | | |
| 1 | randomised trials | not serious^d^ | not serious^a^ | not serious | serious^b^ | none^a^ | mg/dL -0.48 | ⨁◯◯◯ |
|  |  |  |  |  |  |  | (-0.48 to 0.49) | Very low |
| **[5:2] - [CER]** [[54](#_ENREF_54)] | | | | | | | | |
| 6 | randomised trials | not serious | serious^g^ | not serious | serious^b^ | none^e^ | mg/dL -0.2 | ⨁⨁◯◯ |
|  |  |  |  |  |  |  | (-0.37 to 0.02) | Low |
| **[TRE] - [Usual]** [[21](#_ENREF_21)] | | | | | | | | |
| 4 | randomised trials | serious^f^ | very serious^c^ | not serious | serious^b^ | none | mg/dL 9.14 | ⨁◯◯◯ |
|  |  |  |  |  |  |  | (-3.69 to 21.96) | Very low |

**Explanations**

a. only one study

b. small sample size (total population <400)

c. I-square ≥ 75%

d. Only one study was included, and the risk of bias in this study was assessed as high risk.

e. no analysis

f. The included studies with weights over 50% were considered to have a high risk of bias in at least one domain.

g. I-square 40~75%

## (M) Low density lipoprotein cholesterol (LDL-C)

| **Certainty assessment** | | | | | | | **Effect** | **Certainty** |
| --- | --- | --- | --- | --- | --- | --- | --- | --- |
| **№ of studies** | **Study design** | **Risk of bias** | **Inconsistency** | **Indirectness** | **Imprecision** | **Publication bias** | **Relative** |  |
|  |  |  |  |  |  |  | **(95% CI)** |  |
| **[TRE] - [Usual/CER]** [50] | | | | | | | | |
| 10 | randomised trials | not serious | very serious^a^ | not serious | not serious | none | mg/dL -3.63 | ⨁⨁◯◯ |
|  |  |  |  |  |  |  | (-8.05 to 0.78) | Low |
| **[TRE] - [ADF]** [26] | | | | | | | | |
| 1 | randomised trials | not serious | not serious^b^ | not serious | serious^c^ | none^b^ | mg/dL -3.09 | ⨁◯◯◯ |
|  |  |  |  |  |  |  | (-12.55 to 6.37) | Very low |
| **[ADF] - [Usual]** [[25](#_ENREF_25)] | | | | | | | | |
| 5 | randomised trials | serious^g^ | very serious^a^ | not serious | serious^c^ | none | mg/dL -5.82 | ⨁◯◯◯ |
|  |  |  |  |  |  |  | (-10.45 to -1.19) | Very low |
| **[TRE] - [CER]** [[54](#_ENREF_54)] | | | | | | | | |
| 1 | randomised trials | very serious^e^ | not serious^b^ | not serious | serious^c^ | none^b^ | mg/dL -0.07 | ⨁◯◯◯ |
|  |  |  |  |  |  |  | (-0.55 to 0.42) | Very low |
| **[ADF] - [CER]** [[54](#_ENREF_54)] | | | | | | | | |
| 3 | randomised trials | not serious | not serious | not serious | serious^c^ | none^f^ | mg/dL 0.00 | ⨁⨁⨁◯ |
|  |  |  |  |  |  |  | (-0.26 to 0.26) | Moderate |
| **[5:2] - [CER]** [[54](#_ENREF_54)] | | | | | | | | |
| 6 | randomised trials | not serious | not serious | not serious | not serious | none^f^ | mg/dL -0.20 | ⨁⨁⨁⨁ |
|  |  |  |  |  |  |  | (-0.38 to -0.02) | High |
| **[5:2/4:3] - [Usual]** [[37](#_ENREF_37)] | | | | | | | | |
| 5 | randomised trials | not serious | very serious^a^ | not serious | serious^c^ | none | mg/dL -1.44 | ⨁◯◯◯ |
|  |  |  |  |  |  |  | (-5.40 to 2.70) | Very low |
| **[5:2/4:3] - [CER]** [[37](#_ENREF_37)] | | | | | | | | |
| 8 | randomised trials | serious^d^ | not serious | not serious | not serious | none | mg/dL 0.00 | ⨁⨁⨁◯ |
|  |  |  |  |  |  |  | (-1.98 to 1.98) | Moderate |
| **[TRE] - [Usual]** [[21](#_ENREF_21)] | | | | | | | | |
| 4 | randomised trials | serious^d^ | serious^g^ | not serious | serious^c^ | none | mg/dL 3.99 | ⨁◯◯◯ |
|  |  |  |  |  |  |  | (-6.41 to 14.39) | Very low |

**Explanations**

a. I-square ≥ 75%

b. only one study

c. small sample size (total population <400)

d. The included studies with weights over 50% were considered to have a high risk of bias in at least one domain.

e. Only one study was included, and the risk of bias in this study was assessed as high risk.

f. no analysis

g. I-square 40~75%

## (N) High density lipoprotein cholesterol (HDL-C)

| **Certainty assessment** | | | | | | | **Effect** | **Certainty** |
| --- | --- | --- | --- | --- | --- | --- | --- | --- |
| **№ of studies** | **Study design** | **Risk of bias** | **Inconsistency** | **Indirectness** | **Imprecision** | **Publication bias** | **Relative** |  |
|  |  |  |  |  |  |  | **(95% CI)** |  |
| **[TRE] - [Usual/CER]** [50] | | | | | | | | |
| 12 | randomised trials | not serious | serious^f^ | not serious | not serious | none | mg/dL 0.75 | ⨁⨁⨁◯ |
|  |  |  |  |  |  |  | (-0.73 to 2.24) | Moderate |
| **[TRE] - [CER]** [[54](#_ENREF_54)] | | | | | | | | |
| 1 | randomised trials | very serious^b^ | not serious^c^ | not serious | serious^d^ | none^c^ | mg/dL 0.18 | ⨁◯◯◯ |
|  |  |  |  |  |  |  | (-0.31 to 0.66) | Very low |
| **[TRE] - [ADF]** [26] | | | | | | | | |
| 1 | randomised trials | not serious | not serious^c^ | not serious | serious^d^ | none^c^ | mg/dL -0.77 | ⨁◯◯◯ |
|  |  |  |  |  |  |  | (-5.40 to 3.85) | Very low |
| **[ADF] - [Usual]** [[25](#_ENREF_25)] | | | | | | | | |
| 5 | randomised trials | not serious | serious^f^ | not serious | serious^d^ | none | mg/dL -1.05 | ⨁⨁◯◯ |
|  |  |  |  |  |  |  | (-2.92 to 0.83) | Low |
| **[ADF] - [CER]** [[54](#_ENREF_54)] | | | | | | | | |
| 3 | randomised trials | not serious | not serious | not serious | serious^d^ | none^e^ | mg/dL -0.34 | ⨁⨁⨁◯ |
|  |  |  |  |  |  |  | (-0.61 to -0.08) | Moderate |
| **[5:2] - [CER]** [[54](#_ENREF_54)] | | | | | | | | |
| 6 | randomised trials | not serious | not serious | not serious | not serious | none^e^ | mg/dL 0.05 | ⨁⨁⨁⨁ |
|  |  |  |  |  |  |  | (-0.12 to 0.23) | High |
| **[TRE] - [Usual]** [[21](#_ENREF_21)] | | | | | | | | |
| 4 | randomised trials | serious^f^ | serious^f^ | not serious | serious^d^ | none^e^ | mg/dL 1.36 | ⨁◯◯◯ |
|  |  |  |  |  |  |  | (-1.43 to 4.14) | Very low |

**Explanations**

a. I-square ≥ 75%

b. Only one study was included, and the risk of bias in this study was assessed as high risk.

c. only one study

d. small sample size (total population <400)

e. no analysis

f. I-square 40~75%

"[A] - [B]" represents a meta-analysis comparing the A diet with the B diet.

5:2: regular eating for five days and energy restriction for two days per week, ADF: alternate day fasting, CER: continuous energy restriction, TRE: time-restricted eating

# Table S4. Criteria and results of the CINeMA assessment

**CINeMA_Criteria** **for each domain**

1. Within-study bias: Based on the Risk of Bias (RoB 2.0), studies were classified as having no concern, some concern, or major concern regarding bias.

2. Reporting bias: Since each comparison includes fewer than 10 studies, and considering that Egger’s test and funnel plots are not recommended for such cases, these comparisons were flagged as having "some concerns." [67,68]

3. Indirectness: Since the majority of the included population were healthy adults, we would assess comparisons involving clinical populations as having "some concern". [61,66]

4. Imprecision: CINeMA compared the treatment effects included in the 95% confidence interval with the range of equivalence. If the 95% confidence interval extended beyond the region of equivalence opposite to the point estimate of the no effect line, then the treatment effect would be rated as having “major concerns”. If the confidence interval extended but did not go beyond the region of equivalence opposite to the no effect line, it would be assigned a rating of “some concern”. If the confidence interval was entirely on one side of the no effect line or entirely within the region of equivalence, then there was “no concerns”. [66]

5. Heterogeneity: CINeMA compared the treatment effect within the prediction interval with the area of equivalence to determine if the result was consistent with 95% confidence interval (imprecision). When the confidence interval and the prediction interval led to the same conclusion, heterogeneity was “no concerns”. When the conclusion drawn from the confidence interval and the prediction interval differ, heterogeneity was of “some/major concerns”. [66]

6. Incoherence: When the 95% confidence intervals of both direct and indirect evidence were below the equivalence range, it was rated as “no concerns”. When the 95% confidence interval of the indirect evidence crossed the equivalence range but the 95% confidence interval of the direct evidence was completely below the equivalence range, it was rated as “some concern”. When the 95% confidence intervals of both direct and indirect comparisons only shared values below the equivalence range, and the 95% confidence interval of the indirect evidence was within and above the equivalence range, it was consistently rated as “major concern”. [66]

CINeMA considers that if the *p*-value of the global design-by-treatment interaction test was less than 0.05, it indicates a "major concerns." If the *p*-value falls between 0.05 and 0.10, it indicated "some concerns." If the *p*-value was greater than 0.10, it suggested "no concerns." Additionally, if the calculation of interaction test statistics based on treatment design is not possible due to the absence of closed loops in the network, it also signifies a "major problem.". [66]

7. Summarizing judgments across the 6 domains: If the domain was rated as “some concern”, the overall rating will be downgraded by one level. If it was rated as “major concern”, the overall rating would be downgraded by two levels. However, there was an interrelation between "Imprecision," "Heterogeneity," and "Incoherence." Therefore, if one or more domains were rated as "major concern," we would downgrade the overall rating by two levels. If none of the domains were rated as "major concern" but at least one domain was rated as "some concern," we would downgrade the overall rating by one level.

**Equivalent range**

| BW  (kg) | BMI  (kg/m^2^) | FM  (kg) | FFM  (kg) | WC  (cm) | SBP  (mmHg) | DBP  (mmHg) | FBG  (mg/dL) | FI  (μU/mL) | HbA1c  (%) | TG  (mg/dL) | TC  (mg/dL) | LDL  (mg/dL) | HDL  (mg/dL) |
| --- | --- | --- | --- | --- | --- | --- | --- | --- | --- | --- | --- | --- | --- |
| 3.8^a^[69] | 1[70,71] | 2.85^b^[72] | 0.95^b^[72] | 4[73] | 2[74] | 2[74] | 18[75] | 1[76] | 0.5[77] | 88.6[78] | 38.7[79] | 11.6[80] | 1.2[81] |

^a^ BW: Clinically significant weight loss is defined as a reduction of at least 5% from the baseline weight. Since the participants included in the study were mostly overweight or obese, for a person with a height of 166.7 cm, a BMI of 27.5 kg/m^2^, and a weight of 76.4 kg, the clinically significant weight loss would be approximately 3.8 kg [82,83].

^b^FM/FFM: When weight is lost, approximately one-quarter comes from FFM (0.95 kg), while the remaining three-quarters come from FM (2.85 kg) [72].

BMI: body mass index, BW: body weight, DBP: diastolic blood pressure, FBG: fasting blood glucose, FFM: fat free mass, FI: fasting insulin, FM: fat mass, HbA1c: hemoglobin A1c Hemoglobin, HDL: high-density lipoprotein, LDL: low-density lipoprotein, SBP: systolic blood pressure, TC: total cholesterol, TG: Triglyceride, WC: waist circumference

## (A) Body weight (BW)

| Comparison | Number of studies | Within-study bias | Reporting bias | Indirectness | Imprecision | Heterogeneity | Incoherence | Confidence rating |
| --- | --- | --- | --- | --- | --- | --- | --- | --- |
| [5:2]-[CER] | 5 | No concerns | Some concerns | No concerns | No concerns | Some concerns | Major concerns | Very low |
| [ADF]-[CER] | 8 | No concerns | Some concerns | No concerns | No concerns | Some concerns | No concerns | Low |
| [ADF]-[TRE] | 1 | No concerns | Some concerns | No concerns | No concerns | Some concerns | No concerns | Low |
| [ADF]-[Usual diet] | 7 | No concerns | Some concerns | No concerns | No concerns | No concerns | Some concerns | Low |
| [CER]-[TRE] | 3 | No concerns | Some concerns | No concerns | No concerns | Some concerns | Major concerns | Very low |
| [CER]-[Usual diet] | 1 | No concerns | Some concerns | No concerns | No concerns | Some concerns | No concerns | Low |
| [TRE]-[Usual diet] | 5 | No concerns | Some concerns | No concerns | No concerns | Some concerns | Some concerns | Low |
| [5:2]-[ADF] | 0 | No concerns | Some concerns | No concerns | No concerns | Some concerns | Major concerns | Very low |
| [5:2]-[TRE] | 0 | No concerns | Some concerns | No concerns | Some concerns | No concerns | Major concerns | Very low |
| [5:2]-[Usual diet] | 0 | No concerns | Some concerns | No concerns | No concerns | Some concerns | Major concerns | Very low |

## (B) Body mass index (BMI)

| Comparison | Number of studies | Within-study bias | Reporting bias | Indirectness | Imprecision | Heterogeneity | Incoherence | Confidence rating |
| --- | --- | --- | --- | --- | --- | --- | --- | --- |
| [5:2]-[CER] | 5 | No concerns | Some concerns | Some concerns | No concerns | Some concerns | No concerns | Very low |
| [ADF]-[CER] | 5 | No concerns | Some concerns | Some concerns | No concerns | Some concerns | No concerns | Very low |
| [ADF]-[TRE] | 1 | No concerns | Some concerns | Some concerns | No concerns | Some concerns | No concerns | Very low |
| [ADF]-[Usual diet] | 5 | No concerns | Some concerns | No concerns | No concerns | No concerns | No concerns | Moderate |
| [CER]-[TRE] | 4 | No concerns | Some concerns | No concerns | No concerns | Some concerns | No concerns | Low |
| [TRE]-[Usual diet] | 1 | No concerns | Some concerns | Some concerns | No concerns | Some concerns | No concerns | Very low |
| [5:2]-[ADF] | 0 | No concerns | Some concerns | Some concerns | Some concerns | No concerns | No concerns | Very low |
| [5:2]-[TRE] | 0 | No concerns | Some concerns | No concerns | Some concerns | No concerns | No concerns | Low |
| [5:2]-[Usual diet] | 0 | No concerns | Some concerns | No concerns | Some concerns | No concerns | No concerns | Low |
| [CER]-[Usual diet] | 0 | No concerns | Some concerns | No concerns | Some concerns | No concerns | No concerns | Low |

## (C) Fat mass (FM)

| Comparison | Number of studies | Within-study bias | Reporting bias | Indirectness | Imprecision | Heterogeneity | Incoherence | Confidence rating |
| --- | --- | --- | --- | --- | --- | --- | --- | --- |
| [5:2]-[CER] | 5 | No concerns | Some concerns | No concerns | Some concerns | Some concerns | No concerns | Low |
| [ADF]-[CER] | 4 | No concerns | Some concerns | No concerns | Some concerns | Some concerns | No concerns | Low |
| [ADF]-[TRE] | 1 | No concerns | Some concerns | No concerns | Some concerns | Some concerns | No concerns | Low |
| [ADF]-[Usual diet] | 6 | No concerns | Some concerns | No concerns | No concerns | Major concerns | No concerns | Very low |
| [CER]-[TRE] | 2 | No concerns | Some concerns | No concerns | Some concerns | Some concerns | No concerns | Low |
| [TRE]-[Usual diet] | 4 | No concerns | Some concerns | No concerns | Some concerns | Some concerns | No concerns | Low |
| [5:2]-[ADF] | 0 | No concerns | Some concerns | No concerns | Major concerns | No concerns | No concerns | Very low |
| [5:2]-[TRE] | 0 | No concerns | Some concerns | No concerns | Major concerns | No concerns | No concerns | Very low |
| [5:2]-[Usual diet] | 0 | No concerns | Some concerns | No concerns | Some concerns | Some concerns | No concerns | Low |
| [CER]-[Usual diet] | 0 | No concerns | Some concerns | No concerns | Some concerns | Some concerns | No concerns | Low |

## (D) Fat free mass (FFM)

| Comparison | Number of studies | Within-study bias | Reporting bias | Indirectness | Imprecision | Heterogeneity | Incoherence | Confidence rating |
| --- | --- | --- | --- | --- | --- | --- | --- | --- |
| [5:2]-[CER] | 5 | No concerns | Some concerns | No concerns | No concerns | No concerns | Major concerns | Very low |
| [ADF]-[CER] | 5 | No concerns | Some concerns | No concerns | Some concerns | No concerns | Major concerns | Very low |
| [CER]-[TRE] | 2 | No concerns | Some concerns | No concerns | Some concerns | No concerns | Major concerns | Very low |
| [TRE]-[Usual diet] | 4 | No concerns | Some concerns | No concerns | No concerns | Some concerns | Major concerns | Very low |
| [5:2]-[ADF] | 0 | No concerns | Some concerns | No concerns | Some concerns | No concerns | Major concerns | Very low |
| [5:2]-[TRE] | 0 | No concerns | Some concerns | No concerns | Some concerns | No concerns | Major concerns | Very low |
| [5:2]-[Usual diet] | 0 | No concerns | Some concerns | No concerns | Some concerns | No concerns | Major concerns | Very low |
| [ADF]-[TRE] | 0 | No concerns | Some concerns | No concerns | Some concerns | No concerns | Major concerns | Very low |
| [ADF]-[Usual diet] | 0 | No concerns | Some concerns | No concerns | Some concerns | No concerns | Major concerns | Very low |
| [CER]-[Usual diet] | 0 | No concerns | Some concerns | No concerns | Some concerns | No concerns | Major concerns | Very low |

## (E) Waist circumference (WC)

| Comparison | Number of studies | Within-study bias | Reporting bias | Indirectness | Imprecision | Heterogeneity | Incoherence | Confidence rating |
| --- | --- | --- | --- | --- | --- | --- | --- | --- |
| [5:2]-[CER] | 5 | No concerns | Some concerns | No concerns | No concerns | No concerns | No concerns | Moderate |
| [ADF]-[CER] | 2 | No concerns | Some concerns | Some concerns | No concerns | Some concerns | No concerns | Very low |
| [ADF]-[TRE] | 1 | No concerns | Some concerns | Some concerns | No concerns | Some concerns | No concerns | Very low |
| [ADF]-[Usual diet] | 3 | No concerns | Some concerns | No concerns | No concerns | Some concerns | No concerns | Low |
| [CER]-[TRE] | 3 | No concerns | Some concerns | No concerns | No concerns | Some concerns | No concerns | Low |
| [TRE]-[Usual diet] | 1 | No concerns | Some concerns | Some concerns | No concerns | No concerns | No concerns | Low |
| [5:2]-[ADF] | 0 | No concerns | Some concerns | Some concerns | No concerns | Some concerns | No concerns | Very low |
| [5:2]-[TRE] | 0 | No concerns | Some concerns | No concerns | No concerns | Some concerns | No concerns | Low |
| [5:2]-[Usual diet] | 0 | No concerns | Some concerns | No concerns | No concerns | Some concerns | No concerns | Low |
| [CER]-[Usual diet] | 0 | No concerns | Some concerns | Some concerns | No concerns | Some concerns | No concerns | Very low |

## (F) Systolic blood pressure (SBP)

| Comparison | Number of studies | Within-study bias | Reporting bias | Indirectness | Imprecision | Heterogeneity | Incoherence | Confidence rating |
| --- | --- | --- | --- | --- | --- | --- | --- | --- |
| [5:2]-[CER] | 4 | No concerns | Some concerns | No concerns | Some concerns | Some concerns | No concerns | Low |
| [ADF]-[CER] | 3 | Some concerns | Some concerns | No concerns | Some concerns | Some concerns | No concerns | Very low |
| [ADF]-[Usual diet] | 4 | No concerns | Some concerns | No concerns | No concerns | Major concerns | No concerns | Very low |
| [CER]-[TRE] | 4 | No concerns | Some concerns | No concerns | Some concerns | Some concerns | No concerns | Low |
| [TRE]-[Usual diet] | 2 | No concerns | Some concerns | No concerns | Major concerns | No concerns | No concerns | Very low |
| [5:2]-[ADF] | 0 | No concerns | Some concerns | No concerns | Some concerns | Some concerns | No concerns | Low |
| [5:2]-[TRE] | 0 | No concerns | Some concerns | No concerns | Major concerns | No concerns | No concerns | Very low |
| [5:2]-[Usual diet] | 0 | No concerns | Some concerns | No concerns | Major concerns | No concerns | No concerns | Very low |
| [ADF]-[TRE] | 0 | No concerns | Some concerns | No concerns | No concerns | Major concerns | No concerns | Very low |
| [CER]-[Usual diet] | 0 | No concerns | Some concerns | No concerns | Major concerns | No concerns | No concerns | Very low |

## (G) Diastolic blood pressure (DBP)

| Comparison | Number of studies | Within-study bias | Reporting bias | Indirectness | Imprecision | Heterogeneity | Incoherence | Confidence rating |
| --- | --- | --- | --- | --- | --- | --- | --- | --- |
| [5:2]-[CER] | 3 | No concerns | Some concerns | No concerns | Major concerns | No concerns | No concerns | Very low |
| [ADF]-[CER] | 3 | Some concerns | Some concerns | No concerns | Major concerns | No concerns | No concerns | Very low |
| [ADF]-[Usual diet] | 4 | No concerns | Some concerns | No concerns | No concerns | Some concerns | No concerns | Low |
| [CER]-[TRE] | 4 | No concerns | Some concerns | No concerns | Some concerns | No concerns | No concerns | Low |
| [CER]-[Usual diet] | 1 | No concerns | Some concerns | No concerns | Some concerns | Some concerns | No concerns | Low |
| [TRE]-[Usual diet] | 2 | No concerns | Some concerns | No concerns | Major concerns | No concerns | No concerns | Very low |
| [5:2]-[ADF] | 0 | No concerns | Some concerns | No concerns | Major concerns | No concerns | No concerns | Very low |
| [5:2]-[TRE] | 0 | No concerns | Some concerns | No concerns | Some concerns | Some concerns | No concerns | Low |
| [5:2]-[Usual diet] | 0 | No concerns | Some concerns | No concerns | Some concerns | Some concerns | No concerns | Low |
| [ADF]-[TRE] | 0 | No concerns | Some concerns | No concerns | Some concerns | No concerns | No concerns | Low |

## (H) Fasting blood glucose (FBG)

| Comparison | Number of studies | Within-study bias | Reporting bias | Indirectness | Imprecision | Heterogeneity | Incoherence | Confidence rating |
| --- | --- | --- | --- | --- | --- | --- | --- | --- |
| [5:2]-[CER] | 7 | No concerns | Some concerns | No concerns | No concerns | No concerns | No concerns | Moderate |
| [ADF]-[CER] | 3 | No concerns | Some concerns | No concerns | No concerns | No concerns | No concerns | Moderate |
| [ADF]-[TRE] | 1 | No concerns | Some concerns | Some concerns | No concerns | No concerns | No concerns | Low |
| [ADF]-[Usual diet] | 4 | No concerns | Some concerns | No concerns | No concerns | No concerns | No concerns | Moderate |
| [CER]-[TRE] | 1 | No concerns | Some concerns | Some concerns | No concerns | No concerns | No concerns | Low |
| [TRE]-[Usual diet] | 4 | No concerns | Some concerns | No concerns | No concerns | No concerns | No concerns | Moderate |
| [5:2]-[ADF] | 0 | No concerns | Some concerns | No concerns | No concerns | No concerns | No concerns | Moderate |
| [5:2]-[TRE] | 0 | No concerns | Some concerns | No concerns | No concerns | No concerns | No concerns | Moderate |
| [5:2]-[Usual diet] | 0 | No concerns | Some concerns | No concerns | No concerns | No concerns | No concerns | Moderate |
| [CER]-[Usual diet] | 0 | No concerns | Some concerns | No concerns | No concerns | No concerns | No concerns | Moderate |

## (I) Fasting insulin (FI)

| Comparison | Number of studies | Within-study bias | Reporting bias | Indirectness | Imprecision | Heterogeneity | Incoherence | Confidence rating |
| --- | --- | --- | --- | --- | --- | --- | --- | --- |
| [5:2]-[CER] | 4 | No concerns | Some concerns | No concerns | Major concerns | No concerns | No concerns | Very low |
| [ADF]-[CER] | 3 | No concerns | Some concerns | No concerns | Major concerns | No concerns | No concerns | Very low |
| [ADF]-[Usual diet] | 2 | No concerns | Some concerns | No concerns | Major concerns | No concerns | No concerns | Very low |
| [CER]-[TRE] | 1 | No concerns | Some concerns | Some concerns | Major concerns | No concerns | No concerns | Very low |
| [TRE]-[Usual diet] | 3 | No concerns | Some concerns | No concerns | Major concerns | No concerns | No concerns | Very low |
| [5:2]-[ADF] | 0 | No concerns | Some concerns | No concerns | Major concerns | No concerns | No concerns | Very low |
| [5:2]-[TRE] | 0 | No concerns | Some concerns | Some concerns | Major concerns | No concerns | No concerns | Very low |
| [5:2]-[Usual diet] | 0 | No concerns | Some concerns | No concerns | Major concerns | No concerns | No concerns | Very low |
| [ADF]-[TRE] | 0 | No concerns | Some concerns | Some concerns | Major concerns | No concerns | No concerns | Very low |
| [CER]-[Usual diet] | 0 | No concerns | Some concerns | No concerns | Major concerns | No concerns | No concerns | Very low |

## (J) Hemoglobin A1c (HbA1c)

| Comparison | Number of studies | Within-study bias | Reporting bias | Indirectness | Imprecision | Heterogeneity | Incoherence | Confidence rating |
| --- | --- | --- | --- | --- | --- | --- | --- | --- |
| [5:2]-[CER] | 4 | No concerns | Some concerns | Some concerns | No concerns | Major concerns | Major concerns | Very low |
| [CER]-[TRE] | 1 | No concerns | Some concerns | Some concerns | No concerns | Major concerns | Major concerns | Very low |
| [5:2]-[TRE] | 0 | No concerns | Some concerns | Some concerns | Some concerns | Some concerns | Major concerns | Very low |

## (K) Triglycerides (TG)

| Comparison | Number of studies | Within-study bias | Reporting bias | Indirectness | Imprecision | Heterogeneity | Incoherence | Confidence rating |
| --- | --- | --- | --- | --- | --- | --- | --- | --- |
| [5:2]-[CER] | 6 | No concerns | Some concerns | No concerns | No concerns | No concerns | No concerns | Moderate |
| [ADF]-[CER] | 3 | Some concerns | Some concerns | No concerns | No concerns | No concerns | No concerns | Low |
| [ADF]-[TRE] | 1 | No concerns | Some concerns | Some concerns | No concerns | No concerns | No concerns | Low |
| [ADF]-[Usual diet] | 5 | No concerns | Some concerns | No concerns | No concerns | No concerns | No concerns | Moderate |
| [CER]-[TRE] | 1 | No concerns | Some concerns | No concerns | No concerns | No concerns | No concerns | Moderate |
| [TRE]-[Usual diet] | 4 | No concerns | Some concerns | No concerns | No concerns | No concerns | No concerns | Moderate |
| [5:2]-[ADF] | 0 | No concerns | Some concerns | No concerns | No concerns | No concerns | No concerns | Moderate |
| [5:2]-[TRE] | 0 | No concerns | Some concerns | No concerns | No concerns | No concerns | No concerns | Moderate |
| [5:2]-[Usual diet] | 0 | No concerns | Some concerns | No concerns | No concerns | No concerns | No concerns | Moderate |
| [CER]-[Usual diet] | 0 | No concerns | Some concerns | No concerns | No concerns | No concerns | No concerns | Moderate |

## (L) Total cholesterol (TC)

| Comparison | Number of studies | Within-study bias | Reporting bias | Indirectness | Imprecision | Heterogeneity | Incoherence | Confidence rating |
| --- | --- | --- | --- | --- | --- | --- | --- | --- |
| [5:2]-[CER] | 6 | No concerns | Some concerns | No concerns | No concerns | No concerns | No concerns | Moderate |
| [ADF]-[CER] | 3 | No concerns | Some concerns | No concerns | No concerns | No concerns | No concerns | Moderate |
| [ADF]-[TRE] | 1 | No concerns | Some concerns | Some concerns | No concerns | No concerns | No concerns | Low |
| [ADF]-[Usual diet] | 5 | No concerns | Some concerns | No concerns | No concerns | No concerns | No concerns | Moderate |
| [CER]-[TRE] | 1 | No concerns | Some concerns | Some concerns | No concerns | No concerns | No concerns | Low |
| [TRE]-[Usual diet] | 4 | No concerns | Some concerns | No concerns | No concerns | No concerns | No concerns | Moderate |
| [5:2]-[ADF] | 0 | No concerns | Some concerns | No concerns | No concerns | No concerns | No concerns | Moderate |
| [5:2]-[TRE] | 0 | No concerns | Some concerns | No concerns | No concerns | No concerns | No concerns | Moderate |
| [5:2]-[Usual diet] | 0 | No concerns | Some concerns | No concerns | No concerns | No concerns | No concerns | Moderate |
| [CER]-[Usual diet] | 0 | No concerns | Some concerns | No concerns | No concerns | No concerns | No concerns | Moderate |

## (M) Low density lipoprotein cholesterol (LDL-C)

| Comparison | Number of studies | Within-study bias | Reporting bias | Indirectness | Imprecision | Heterogeneity | Incoherence | Confidence rating |
| --- | --- | --- | --- | --- | --- | --- | --- | --- |
| [5:2]-[CER] | 6 | No concerns | Some concerns | No concerns | No concerns | Some concerns | No concerns | Low |
| [ADF]-[CER] | 3 | No concerns | Some concerns | No concerns | No concerns | Some concerns | No concerns | Low |
| [ADF]-[TRE] | 1 | No concerns | Some concerns | No concerns | No concerns | Some concerns | No concerns | Low |
| [ADF]-[Usual diet] | 4 | No concerns | Some concerns | No concerns | No concerns | Some concerns | No concerns | Low |
| [CER]-[TRE] | 1 | No concerns | Some concerns | Some concerns | No concerns | Some concerns | No concerns | Very low |
| [CER]-[Usual diet] | 1 | No concerns | Some concerns | No concerns | No concerns | Some concerns | No concerns | Low |
| [TRE]-[Usual diet] | 4 | No concerns | Some concerns | No concerns | No concerns | Some concerns | No concerns | Low |
| [5:2]-[ADF] | 0 | No concerns | Some concerns | No concerns | No concerns | Some concerns | No concerns | Low |
| [5:2]-[TRE] | 0 | No concerns | Some concerns | No concerns | Some concerns | No concerns | No concerns | Low |
| [5:2]-[Usual diet] | 0 | No concerns | Some concerns | No concerns | Some concerns | No concerns | No concerns | Low |

## (N) High density lipoprotein cholesterol (HDL-C)

| Comparison | Number of studies | Within-study bias | Reporting bias | Indirectness | Imprecision | Heterogeneity | Incoherence | Confidence rating |
| --- | --- | --- | --- | --- | --- | --- | --- | --- |
| [5:2]-[CER] | 6 | No concerns | Some concerns | No concerns | Major concerns | No concerns | No concerns | Very low |
| [ADF]-[CER] | 3 | Some concerns | Some concerns | No concerns | No concerns | Some concerns | No concerns | Very low |
| [ADF]-[TRE] | 1 | No concerns | Some concerns | No concerns | No concerns | Some concerns | No concerns | Low |
| [ADF]-[Usual diet] | 5 | No concerns | Some concerns | No concerns | Some concerns | Some concerns | No concerns | Low |
| [CER]-[TRE] | 1 | No concerns | Some concerns | Some concerns | Major concerns | No concerns | No concerns | Very low |
| [CER]-[Usual diet] | 1 | No concerns | Some concerns | No concerns | Some concerns | Some concerns | No concerns | Low |
| [TRE]-[Usual diet] | 4 | No concerns | Some concerns | No concerns | Some concerns | Some concerns | No concerns | Low |
| [5:2]-[ADF] | 0 | No concerns | Some concerns | No concerns | No concerns | Some concerns | No concerns | Low |
| [5:2]-[TRE] | 0 | No concerns | Some concerns | No concerns | Major concerns | No concerns | No concerns | Very low |
| [5:2]-[Usual diet] | 0 | No concerns | Some concerns | No concerns | Some concerns | Some concerns | No concerns | Low |

5:2: regular eating for five days and energy restriction for two days per week, ADF: alternate day fasting, CER: continuous energy restriction, TRE: time-restricted eating

# Table S5. Characteristics of original studies within included studies

| Study | Age (year) | Sex (F%) | No. of participants | Disease | BMI | Intervention duration (weeks) | Comparisons | Outcomes |
| --- | --- | --- | --- | --- | --- | --- | --- | --- |
| Beaulieu 2021 [86] | 35 | 100 | 46 | NA | 29.1 | 12 | [ADF] - [CER] | BW |
| Gray 2021 [87] | 39.6 | 100 | 121 | GDM | 32.6 | 48 | [5:2] - [CER] | BMI, FBG, FI, HbA1c |
| Pureza 2021 [88] | 31.4 | 100 | 58 | NA | 33.3 | 48 | [TRE] - [CER] | BW, BMI, WC, SBP, DBP |
| Steger 2021 [89] | 45.6 | 76.9 | 35 | NA | 31.2 | 12 | [ADF] - [CER] | BW, FFM, DBP |
| Templeman 2021 [90] | 42.4 | 58.3 | 36 | NA | 23.9 | 3 | [ADF] - [CER] | BW |
| Beaulieu 2020 [91] | 34.5 | 100 | 30 | NA | 29.2 | 12 | [ADF] - [CER] | BMI, FM, WC |
| Kunduraci 2020 [92] | 48.1 | 0 | 65 | Metabolic syndrome | 34.7 | 12 | [TRE] - [CER] | BW, BMI, FM, FFM, SBP, DBP,FBG, FI, HbA1c, TG, TC, LDL, HDL |
| Lowe 2020 [93] | 46.5 | 39.7 | 116 | NA | 32.1 | 12 | [TRE] - [CER] | BW, BMI, FM, FFM, WC, SBP, DBP |
| Pureza 2020 [94] | 31 | 100 | 58 | NA | 33.3 | 3 | [TRE] - [CER] | BMI, WC, SBP, DBP |
| Razavi 2020 [95] | 42.2 | 37.3 | 80 | Metabolic syndrome | NA | 16 | [ADF] - [CER] | FFM |
| Gabel 2019 [96] | 42.4 | 78.6 | 28 | Insulin resistant | 36.6 | 48 | [ADF] - [CER] | BW, BMI, FM, FFM, SBP, DBP, FBG, FI, HbA1c, TG, TC, LDL, HDL |
| Headland 2019 [97] | 49.5 | 82 | 102 | NA | 33.5 | 48 | [5:2] - [CER] | BMI, FM, FFM, FBG, TG, TC, LDL, HDL |
| Cai 2019 [99] | 34.5 | 0 | 264 | NAFLD | 26.4 | 12 | [TRE] - [ADF] - [Usual] | BW, BMI, FM, WC, FBG, FI, TG, LDL, HDL |
| Carter 2019 [99] | 61 | 56 | 97 | T2DM | 36 | 48 | [5:2] - [CER] | FM, FFM |
| Cho 2019 [100] | 37 | 61.5 | 13 | NA | 27.03 | 8 | [ADF] - [Usual] | BW, BMI, FM, FBG, TG, TC, LDL, HDL |
| Hutchison 2019 [101] | 50 | 100 | 51 | NA | 32.3 | 8 | [ADF] - [CER] | BW |
| Johari 2019 [102] | 47 | 30 | 43 | NAFLD | 30.8 | 8 | [ADF] - [Usual] | BW, FBG, TC, LDL, HDL, TG |
| Parvaresh 2019 [103] | 45.5 | 40.6 | 69 | Metabolic syndrome | 31.3 | 8 | [ADF] - [CER] | BMI, WC, SBP, FBG, FI, TG, TC |
| Stekovic 2019 [104] | 49.2 | 72.3 | 57 | NA | 25.2 | 4 | [ADF] - [Usual] | BW, BMI, FM, SBP, DBP |
| Bowen 2018 [105] | 40.3 | 81 | 163 | NA | 35.6 | 24 | [ADF] - [CER] | BW, BMI, FM, FFM, SBP, DBP, FBG, FI, TG, TC, LDL, HDL |
| Carter 2018 [106] | 61 | 56.2 | 137 | T2DM | 36 | 48 | [5:2] - [CER] | BMI, HbA1c |
| Conley 2018 [107] | 67.5 | 0 | 23 | NA | 34.9 | 24 | [5:2] - [CER] | BW, BMI, WC, SBP, DBP, FBG, TG, TC, LDL, HDL |
| Coutinho 2018 [108] | 39 | 79 | 28 | NA | 35.4 | 12 | [ADF] - [CER] | BW, BMI, FM, FFM, SBP |
| Oh 2018 [109] | 36.2 | 69.6 | 23 | NA | 27.03 | 8 | [ADF] - [Usual] | BW, BMI, FM, WC, SBP, DBP, FBG, TG, TC, HDL |
| Schübel 2018 [110] | 50 | 49 | 98 | NA | 31.6 | 12 | [5:2] - [CER] | BW, WC, FBG, FI, HbA1c, TG, TC, LDL, HDL |
| Sundfør 2018 [111] | 48.7 | 50 | 112 | Metabolic syndrome | 35.2 | 24 | [5:2] - [CER] | BW, BMI, WC, SBP, DBP, FBG, TG, TC, LDL, HDL |
| Sutton 2018 [112] | 56 | 0 | 8 | preDM | 32.2 | 5.3 | [TRE] - [Usual] | BW, SBP, DBP, FBG, FI, TG, TC, LDL, HDL |
| Barnosky 2017 [113] | 42.2 | 10.5 | 38 | NA | 34 | 24 | [ADF] - [Usual] | BW, FM |
| Tinsley 2017 [114] | 22.5 | 0 | 8 | NA | 25.7 | 8 | [TRE] - [Usual] | BW, FM, FFM |
| Trepanowski 2017 [115] | 44 | 87.7 | 65 | NA | 35 | 24 | [ADF] - [Usual] | BW, FM, FFM, SBP, DBP, FBG, TG, TC, LDL, HDL |
| Carter 2016 [116] | 61.5 | 52.4 | 63 | T2DM | 35.2 | 48 | [5:2] - [CER] | FM, FFM |
| Catenacci 2016 [117] | 41.1 | 76 | 21 | NA | 37.6 | 8 | [ADF] - [CER] | BW, BMI, FM, FBG, FI, TG, TC |
| Moro 2016 [118] | 29.2 | 0 | 34 | NA | 26.5 | 8 | [TRE] - [Usual] | BW, FM, FFM, FBG, FI, TG, TC, LDL, HDL |
| Betts 2014 [119] | 36 | 64 | 33 | NA | 22.4 | 6 | [TRE] - [Usual] | BW, FM, FFM, FBG, FI, TG, TC, LDL, HDL |
| Bhutani 2013 [120] | 45.5 | 95 | 32 | NA | 35 | 12 | [ADF] - [Usual] | BMI, WC, FI |
| Harvie 2013 [121] | 46.8 | 100 | 77 | NA | 31 | 12 | [5:2] - [CER] | BW, FM, FFM, WC, SBP, FBG, FI, HbA1c, TG, TC, LDL, HDL |
| Varady 2013 [122] | 47.5 | 73 | 30 | NA | 26 | 12 | [ADF] - [Usual] | BW, FM, SBP, DBP, TG, TC, LDL, HDL |
| Harvie 2011 [123] | 40 | 100 | 107 | NA | 30.6 | 24 | [5:2] - [CER] | BW, FM, FFM, WC, SBP, DBP, FBG, FI, TG, TC, LDL, HDL |
| Varady 2011 [124] | 47 | 80 | 30 | NA | 32 | 12 | [ADF] - [CER] | LDL, HDL |
| Stote 2007 [125] | 45 | 66 | 15 | NA | 23.4 | 8 | [TRE] - [Usual] | BW, FM, FFM, SBP, DBP, FBG, TG, TC, LDL, HDL |

5:2: regular eating for five days and energy restriction for two days per week, ADF: alternate day fasting, BMI: body mass index, BW: body weight, CER: continuous energy restriction, DBP: diastolic blood pressure, FBG: fasting blood glucose, GDM: gestational diabetes mellitus, FFM: fat free mass, FI: fasting insulin, FM: Fat mass, HbA1c: hemoglobin A1c, HDL-C: high density lipoprotein cholesterol, IF: intermittent fasting, LDL-C: low density lipoprotein cholesterol, NAFLD: nonalcoholic fatty liver disease, preDM: prediabetes mellitus, SBP: systolic blood pressure, T2DM: type 2 diabetes mellitus, TC: Total cholesterol, TG: triglycerides, TRE: time-restricted eating, WC: waist circumference

# Table S6. Network evidence of pairs-wise comparison with CINeMA

## (A) Body weight (BW)

| Usual | **-3.22**  **(-4.63,-1.81)** | **-3.72**  **(-6.10,-1.35)** | **-3.82**  **(-4.87,-2.77)** | **-2.02**  **(-3.52,-0.53)** |
| --- | --- | --- | --- | --- |
|  | CER | -0.50  (-2.43,1.43) | -0.60  (-1.72,0.52) | 1.20  (-0.52,2.91) |
|  |  | 5:2 | -0.10  (-2.32,2.13) | 1.70  (-0.87,4.27) |
|  |  |  | ADF | **1.79**  **(0.21,3.38)** |
|  |  |  |  | TRE |

## (B) Body mass index (BMI)

| Usual | -0.40 (-1.08,0.28) | -0.21 (-1.10,0.68) | **-0.93 (-1.36,-0.49)** | **-0.79 (-1.51,-0.08)** |
| --- | --- | --- | --- | --- |
|  | CER | 0.19 (-0.37,0.75) | -0.52 (-1.07,0.02) | -0.39 (-1.00,0.22) |
|  |  | 5:2 | -0.71 (-1.50,0.08) | -0.58 (-1.41,0.26) |
|  |  |  | ADF | 0.13 (-0.51,0.78) |
|  |  |  |  | TRE |

## (C) Fat mass (FM)

| Usual | -3.34 (-6.89,0.21) | -3.74 (-8.48,1.00) | **-4.29 (-6.65,-1.93)** | -2.39 (-5.32,0.53) |
| --- | --- | --- | --- | --- |
|  | CER | -0.40 (-3.54,2.74) | -0.95 (-4.00,2.10) | 0.95 (-2.58,4.47) |
|  |  | 5:2 | -0.55 (-4.92,3.83) | 1.35 (-3.37,6.06) |
|  |  |  | ADF | 1.89 (-1.22,5.00) |
|  |  |  |  | TRE |

## (D) Fat free mass/Lean mass (FFM/LM)

| Usual | 0.81  (-0.73,2.34) | 0.60  (-1.10,2.29) | 1.23  (-0.59,3.06) | 0.04  (-1.01,1.09) |
| --- | --- | --- | --- | --- |
|  | CER | -0.21  (-0.93,0.51) | 0.43  (-0.56,1.41) | -0.77  (-1.88,0.35) |
|  |  | 5:2 | 0.64  (-0.58,1.86) | -0.55  (-1.88,0.77) |
|  |  |  | ADF | -1.19  (-2.68,0.30) |
|  |  |  |  | TRE |

## (E) Waist circumference (WC)

| Usual | 1.43  (-1.07,3.93) | 1.00  (-2.09,4.10) | -1.72  (-3.49,0.04) | 0.06  (-2.19,2.31) |
| --- | --- | --- | --- | --- |
|  | CER | -0.43  (-2.18,1.32) | **-3.15  (-5.20,-1.10)** | -1.37  (-3.31,0.57) |
|  |  | 5:2 | -2.72  (-5.46,0.01) | -0.94  (-3.57,1.68) |
|  |  |  | ADF | 1.78  (-0.22,3.79) |
|  |  |  |  | TRE |

## (F) Systolic blood pressure (SBP)

| Usual | -4.01  (-8.64,0.62) | -1.22  (-7.56,5.12) | **-5.00  (-7.97,-2.04)** | -2.79  (-7.70,2.12) |
| --- | --- | --- | --- | --- |
|  | CER | 2.79  (-1.48,7.07) | -0.99  (-5.14,3.16) | 1.22  (-2.61,5.06) |
|  |  | 5:2 | -3.78  (-9.79,2.22) | -1.57  (-7.31,4.17) |
|  |  |  | ADF | 2.21  (-2.67,7.10) |
|  |  |  |  | TRE |

## (G) Diastolic blood pressure (DBP)

| Usual | **-3.53  (-7.06,-0.00)** | -3.92  (-8.64,0.80) | **-3.38  (-5.68,-1.08)** | -1.71  (-5.62,2.20) |
| --- | --- | --- | --- | --- |
|  | CER | -0.39  (-3.54,2.76) | 0.15  (-2.93,3.23) | 1.82  (-1.14,4.78) |
|  |  | 5:2 | 0.53  (-3.87,4.94) | 2.21  (-2.12,6.53) |
|  |  |  | ADF | 1.67  (-2.10,5.44) |
|  |  |  |  | TRE |

## (H) Fasting blood glucose (FBG)

| Usual | -2.03  (-6.79,2.73) | -1.07  (-6.46,4.32) | -2.71  (-5.74,0.32) | -2.52  (-6.15,1.11) |
| --- | --- | --- | --- | --- |
|  | CER | 0.96  (-1.57,3.49) | -0.68  (-4.48,3.13) | -0.49  (-5.90,4.92) |
|  |  | 5:2 | -1.64  (-6.21,2.93) | -1.45  (-7.42,4.52) |
|  |  |  | ADF | 0.19  (-3.97,4.35) |
|  |  |  |  | TRE |

## (I) Fasting insulin (FI)

| Usual | -1.45  (-4.43,1.52) | -2.04  (-5.39,1.31) | -1.54  (-4.17,1.08) | -1.02  (-2.78,0.74) |
| --- | --- | --- | --- | --- |
|  | CER | -0.59  (-2.13,0.96) | -0.09  (-2.21,2.03) | 0.43  (-2.64,3.51) |
|  |  | 5:2 | 0.50  (-2.13,3.13) | 1.02  (-2.42,4.46) |
|  |  |  | ADF | 0.52  (-2.40,3.45) |
|  |  |  |  | TRE |

## (J) Hemoglobin A1c (HbA1c)

| CER | 0.02  (-0.15,0.19) | -0.01  (-0.44,0.42) |
| --- | --- | --- |
|  | 5:2 | -0.03  (-0.48,0.43) |
|  |  | TRE |

## (K) Triglycerides (TG)

| Usual | -3.10  (-27.01,20.81) | -1.23  (-29.39,26.93) | -8.36  (-21.47,4.74) | 0.08  (-15.12,15.28) |
| --- | --- | --- | --- | --- |
|  | CER | 1.87  (-12.93,16.67) | -5.26  (-26.25,15.73) | 3.19  (-22.05,28.43) |
|  |  | 5:2 | -7.13  (-32.87,18.61) | 1.32  (-27.95,30.58) |
|  |  |  | ADF | 8.45  (-8.96,25.86) |
|  |  |  |  | TRE |

## (L) Total cholesterol (TC)

| Usual | -4.08  (-15.30,7.15) | -9.08  (-22.40,4.24) | **-10.54  (-17.08,-3.99)** | 5.55  (-2.08,13.17) |
| --- | --- | --- | --- | --- |
|  | CER | -5.01  (-12.18,2.17) | -6.46  (-16.60,3.68) | 9.62  (-1.98,21.23) |
|  |  | 5:2 | -1.46  (-13.88,10.96) | **14.63  (0.99,28.27)** |
|  |  |  | ADF | **16.09  (7.11,25.06)** |
|  |  |  |  | TRE |

##

## (M) Low density lipoprotein cholesterol (LDL-C)

| Usual | -3.18  (-9.25,2.89) | **-7.37  (-14.74,-0.00)** | **-4.63  (-7.92,-1.34)** | -1.44  (-6.69,3.81) |
| --- | --- | --- | --- | --- |
|  | CER | **-4.19  (-8.37,-0.01)** | -1.45  (-7.07,4.17) | 1.74  (-4.72,8.20) |
|  |  | 5:2 | 2.74  (-4.26,9.74) | 5.93  (-1.77,13.62) |
|  |  |  | ADF | 3.19  (-2.37,8.75) |
|  |  |  |  | TRE |

## (N) High density lipoprotein cholesterol (HDL-C)

| Usual | 0.95  (-1.61,3.51) | 1.43  (-1.68,4.54) | -1.25  (-2.71,0.22) | 0.83  (-1.13,2.79) |
| --- | --- | --- | --- | --- |
|  | CER | 0.48  (-1.31,2.27) | -2.20  (-4.55,0.15) | -0.12  (-2.72,2.48) |
|  |  | 5:2 | -2.68  (-5.62,0.27) | -0.60  (-3.74,2.54) |
|  |  |  | ADF | 2.08  (-0.09,4.25) |
|  |  |  |  | TRE |


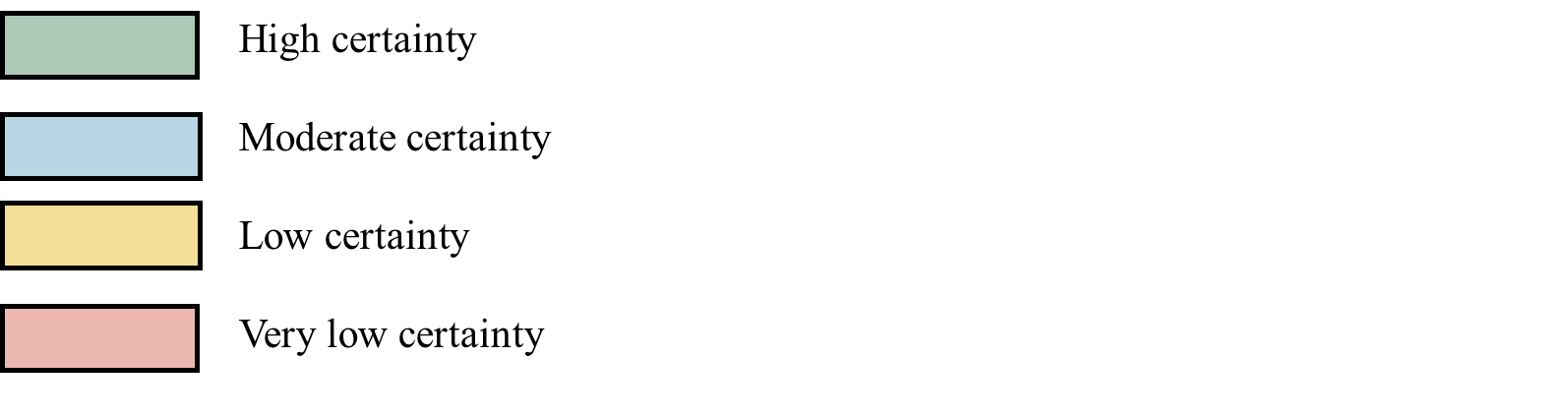


5:2: regular eating for five days and energy restriction for two days per week, ADF: alternate day fasting, CER: continuous energy restriction, TRE: time-restricted eating

# Table S7. Results for meta-regression

| Outcomes | Meta regression (*p*-value) | | | |
| --- | --- | --- | --- | --- |
|  | BMI | Age | Gender | Duration of intervention |
| Body weight (BW) | **0.0116** | 0.9162 | 0.9305 | **0.0003** |
| Body mass index (BMI) | 0.4540 | 0.2991 | 0.2538 | 0.3372 |
| Fat mass (FM) | **0.0158** | 0.9370 | 0.1348 | **0.0020** |
| Fat-free mass/lean mass (FFM/LM) | 0.9912 | 0.9257 | 0.9476 | 0.9985 |
| Waist circumference (WC) | **0.0040** | **0.0185** | 0.8536 | 0.2848 |
| Systolic blood pressure (SBP) | 0.3953 | 0.4003 | 0.1507 | 0.9909 |
| Diastolic blood pressure (DBP) | 0.7313 | 0.4281 | 0.2685 | 0.8551 |
| Fasting blood glucose (FBG) | 0.5786 | 0.5368 | 0.5431 | 0.8571 |
| Fasting insulin (FI) | 0.7906 | 0.1249 | **0.0001*** | 0.6896 |
| Hemoglobin A1c (HbA1c) | 0.1220 | 0.5863 | 0.4413 | 0.1329 |
| Total triglycerides (TG) | 0.3658 | 0.0456 | 0.4536 | 0.1479 |
| Total cholesterol (TC) | 0.2395 | 0.0393 | 0.5394 | 0.8340 |
| Low-density-lipoprotein-cholesterol (LDL-C) | 0.7271 | 0.5680 | 0.1343 | 0.6771 |
| High-density-lipoprotein-cholesterol (HDL-C) | 0.1768 | 0.4729 | 0.9537 | 0.6869 |

*Despite significant *p*-value based on likelihood ratio test, no significant differences in treatment effects according to gender were identified for all IF compared to a usual diet.

BMI: body mass index

# Figure S1. Summary of the direct evidence

## (A) Body weight (BW)


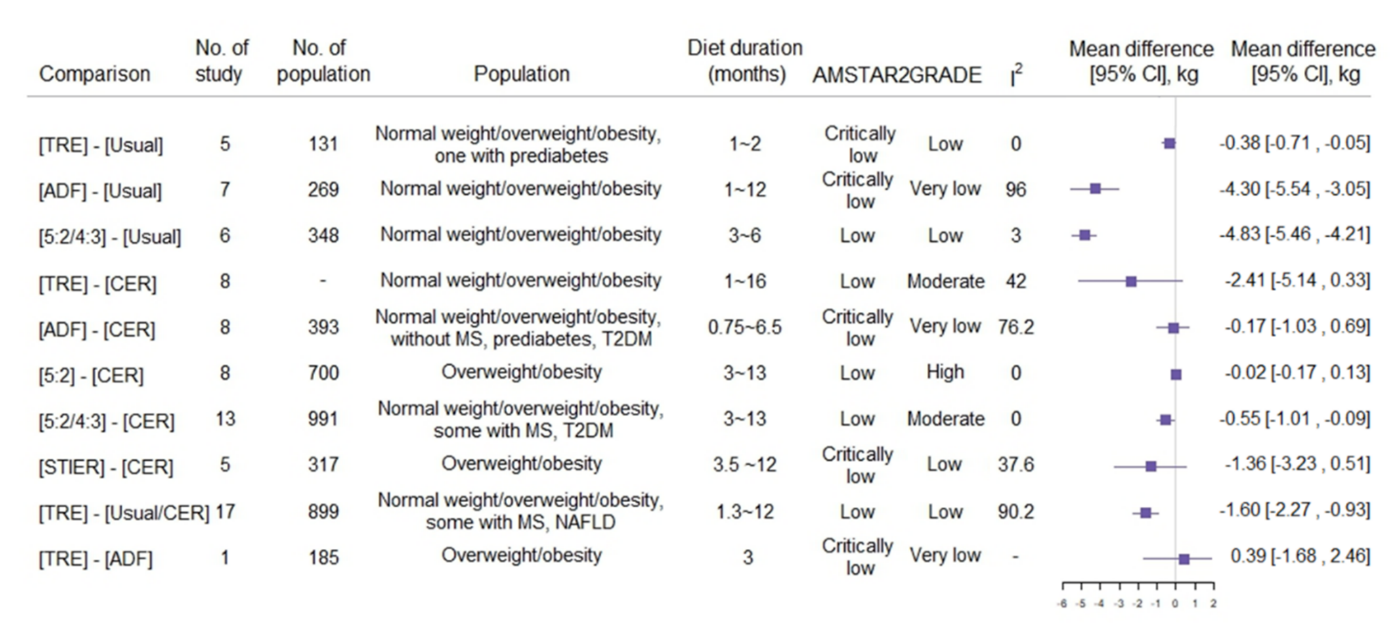


## (B) Body mass index (BMI)


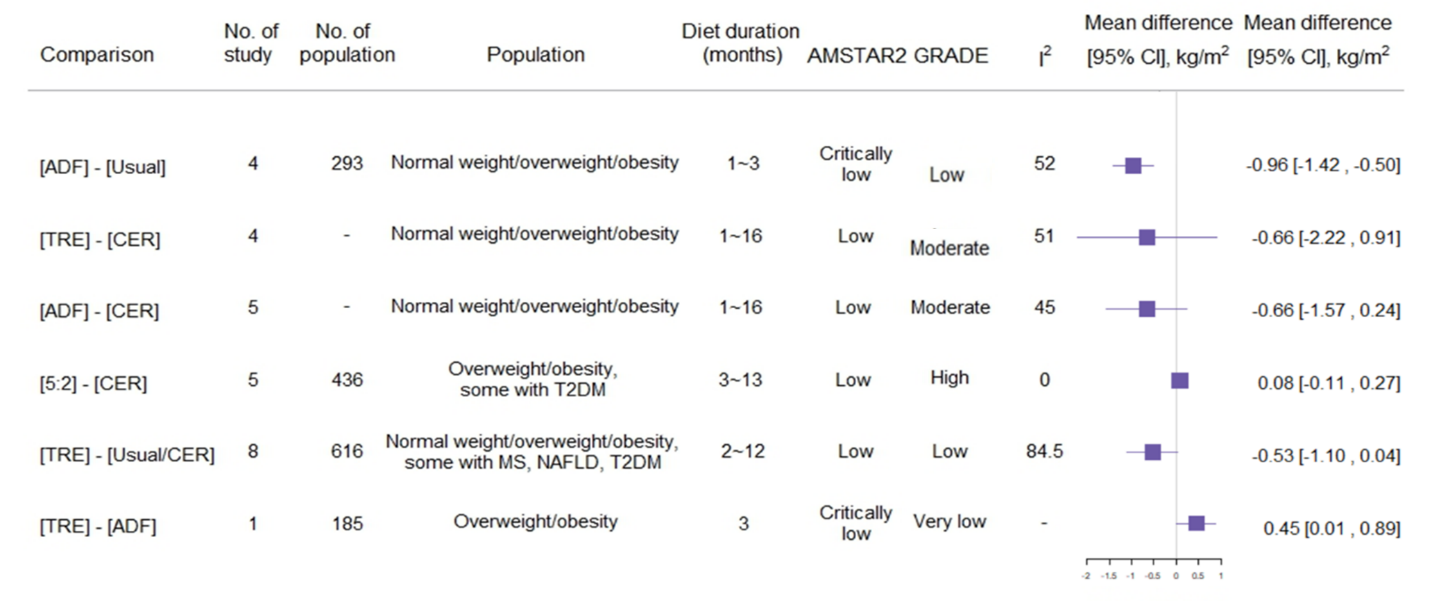


## (C) Fat mass (FM)


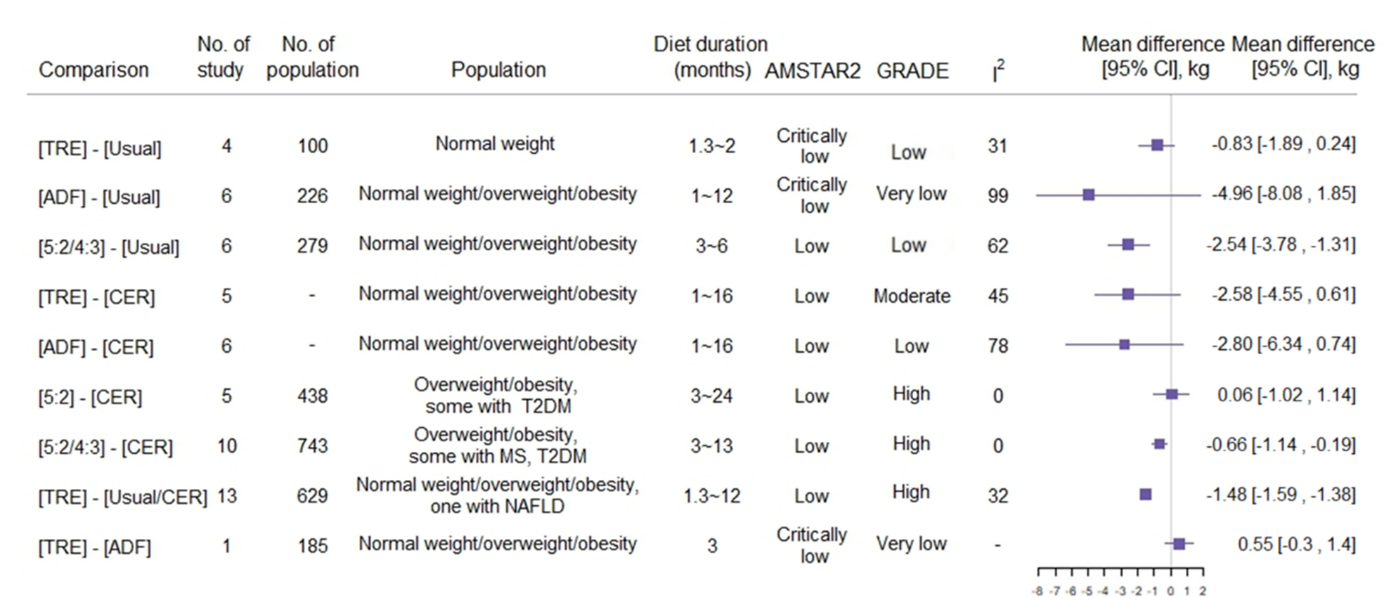


## (D) Fat free mass (FFM)


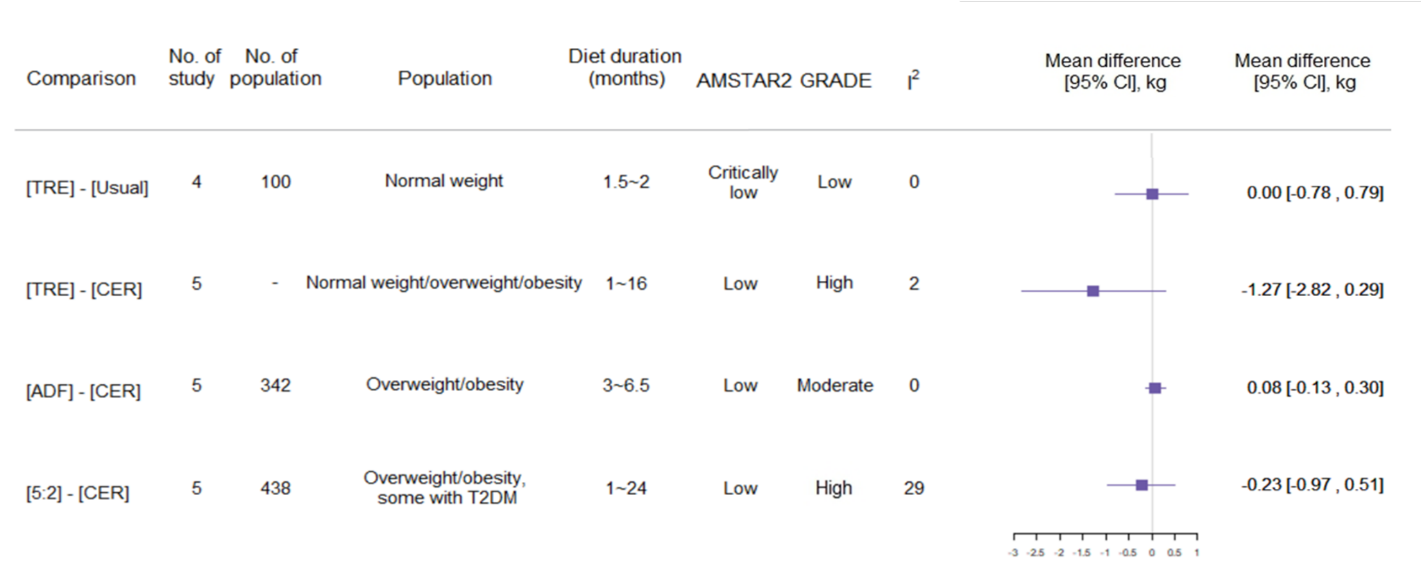


## (E) Waist circumference (WC)


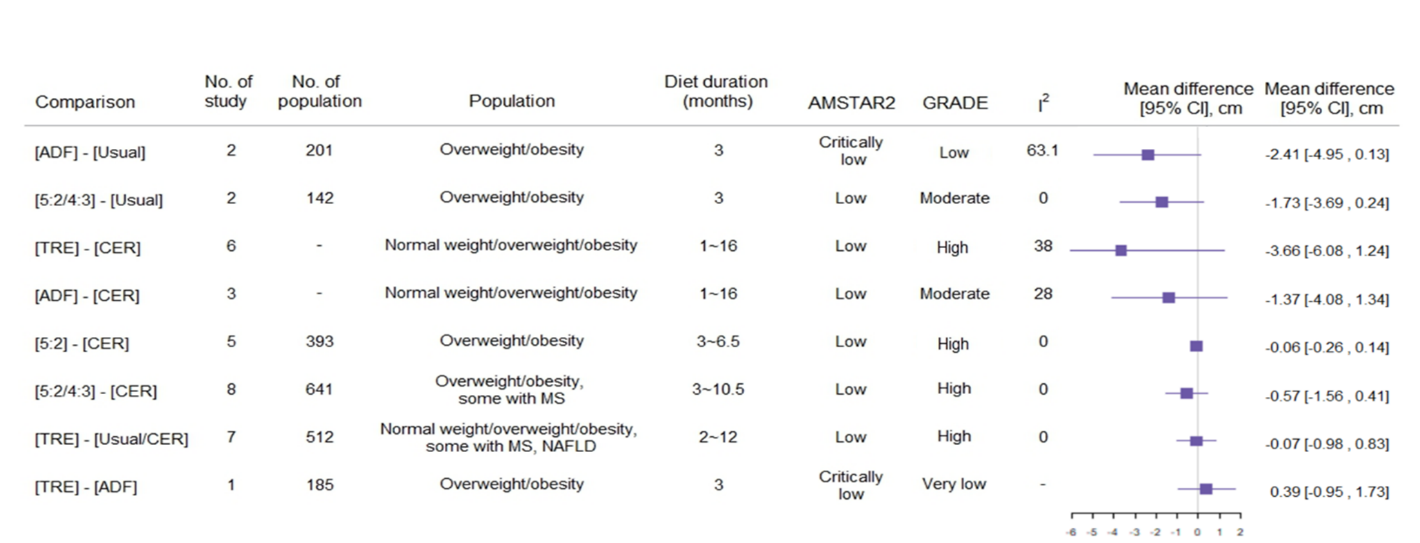


## (F) Systolic blood pressure (SBP)


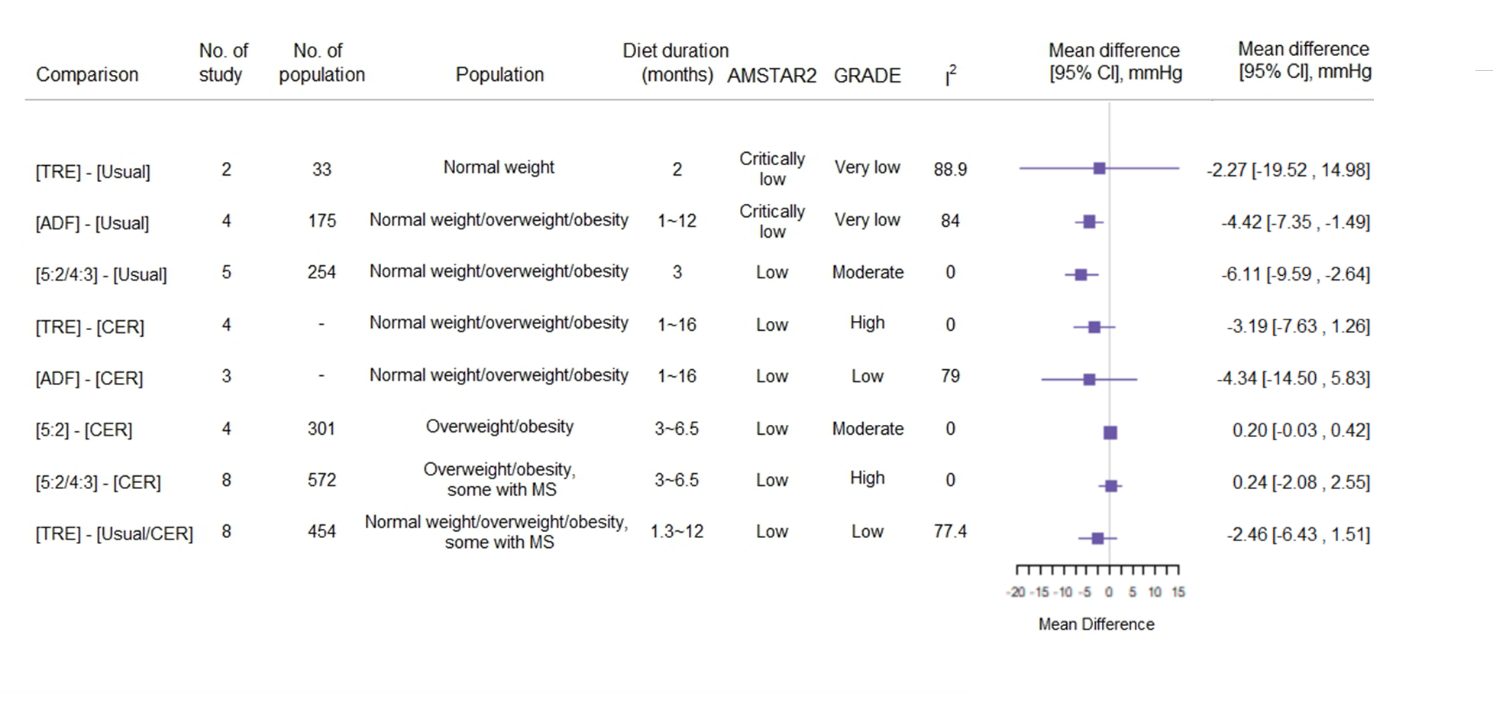


## (G) Diastolic blood pressure (DBP)


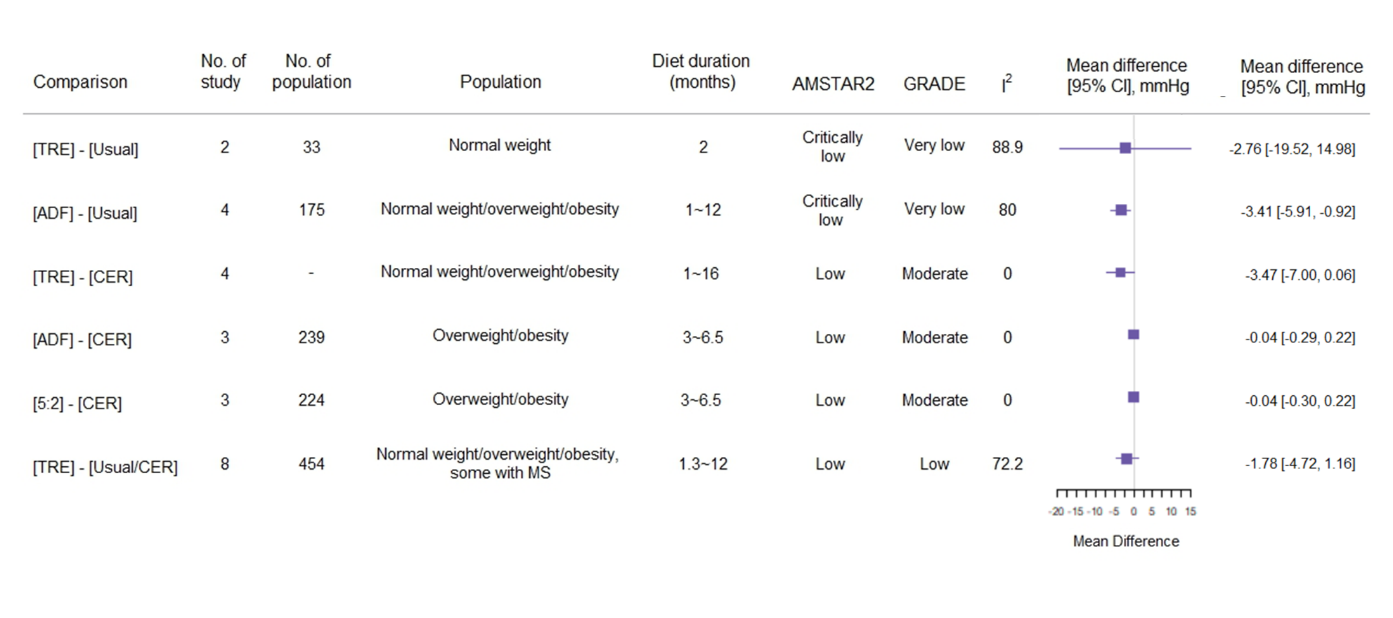


## (H) Fasting blood glucose (FBG)


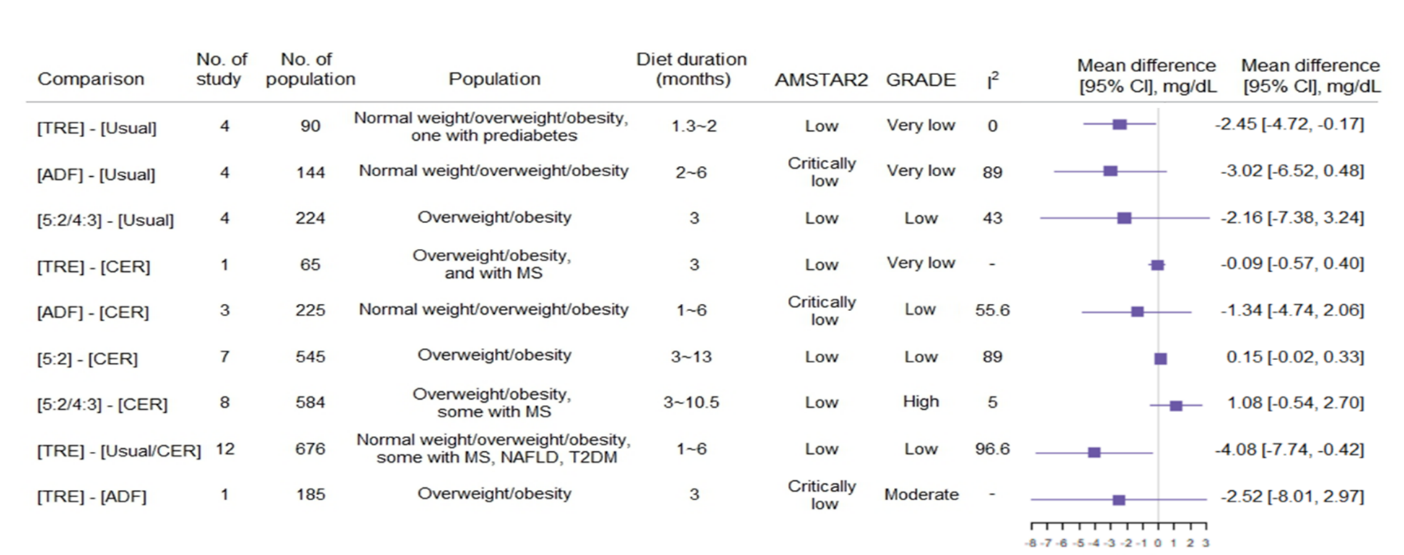


(I) Fasting insulin (FI)


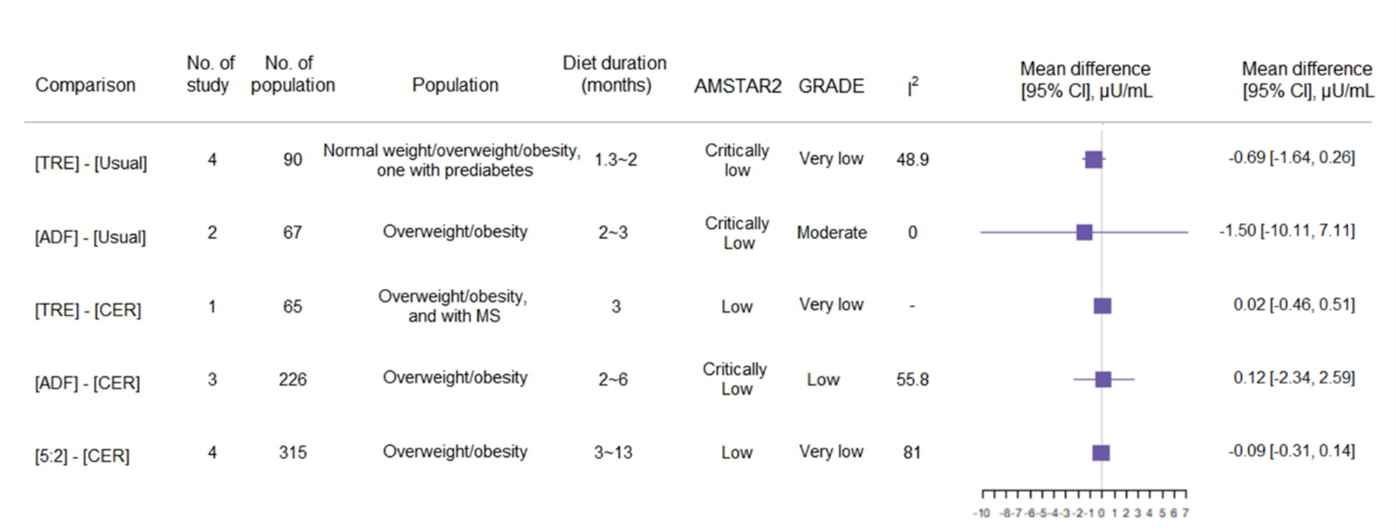


## (J) Hemoglobin A1c (HbA1c)


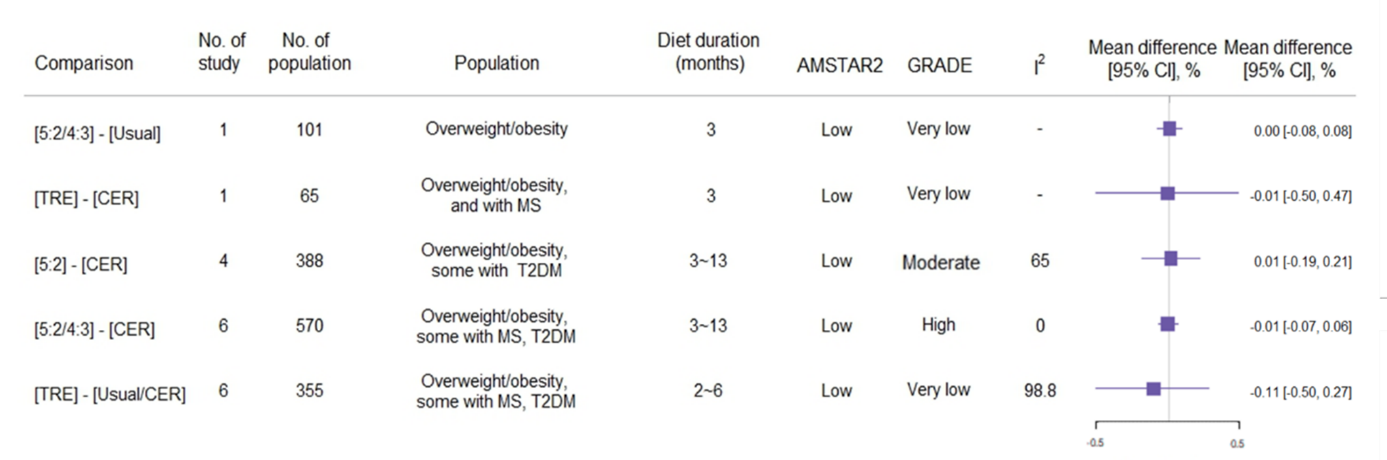


## (K) Triglycerides (TG)


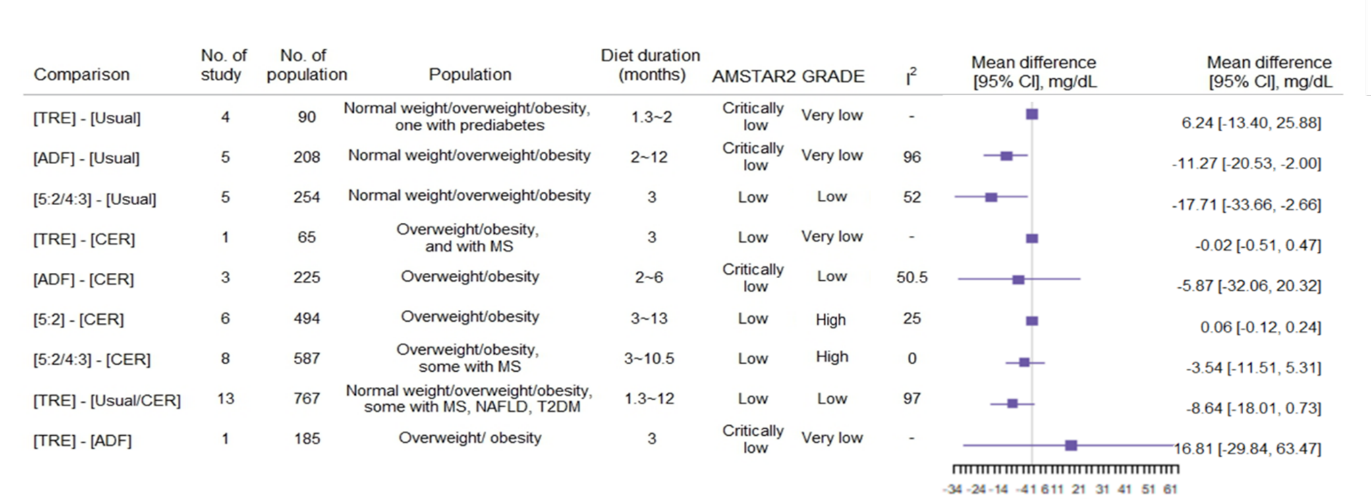


## (L) Total cholesterol (TC)


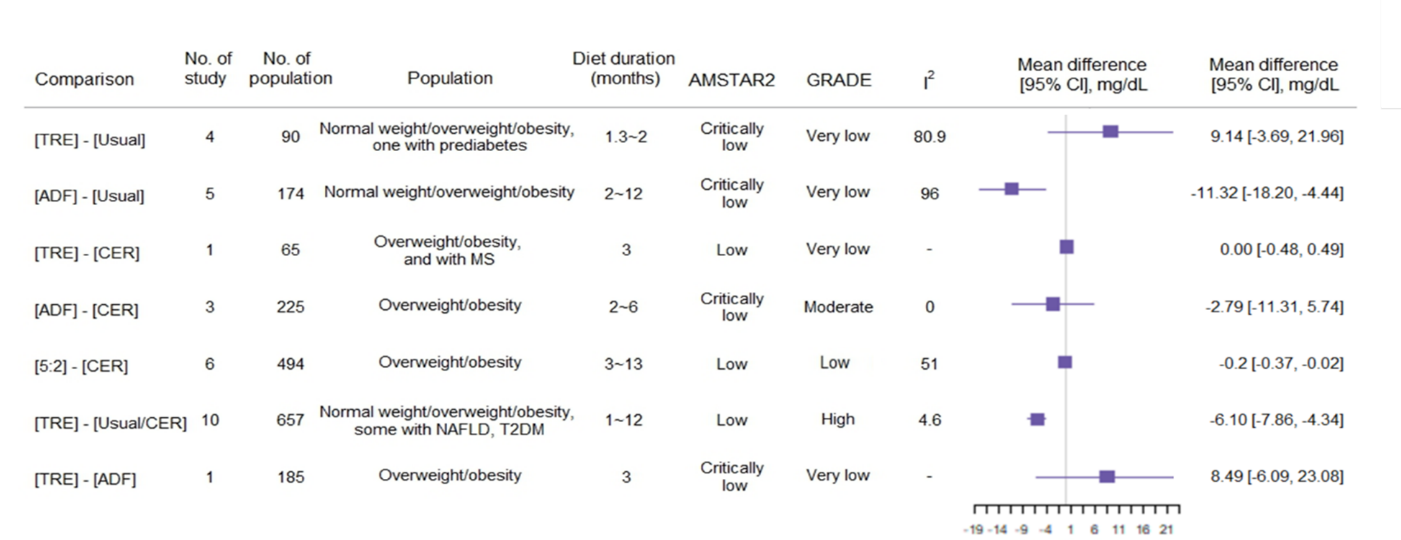


## (M) Low density lipoprotein cholesterol (LDL-C)


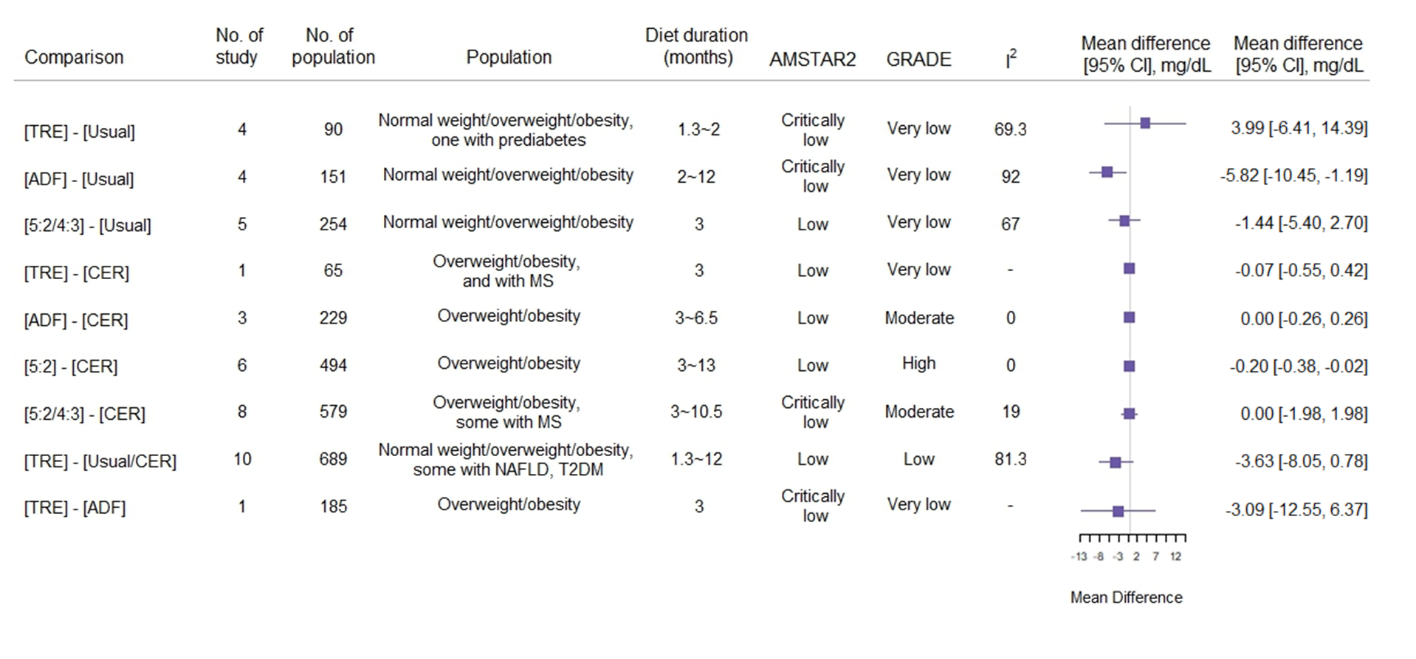


## (N) High density lipoprotein cholesterol (HDL-C)


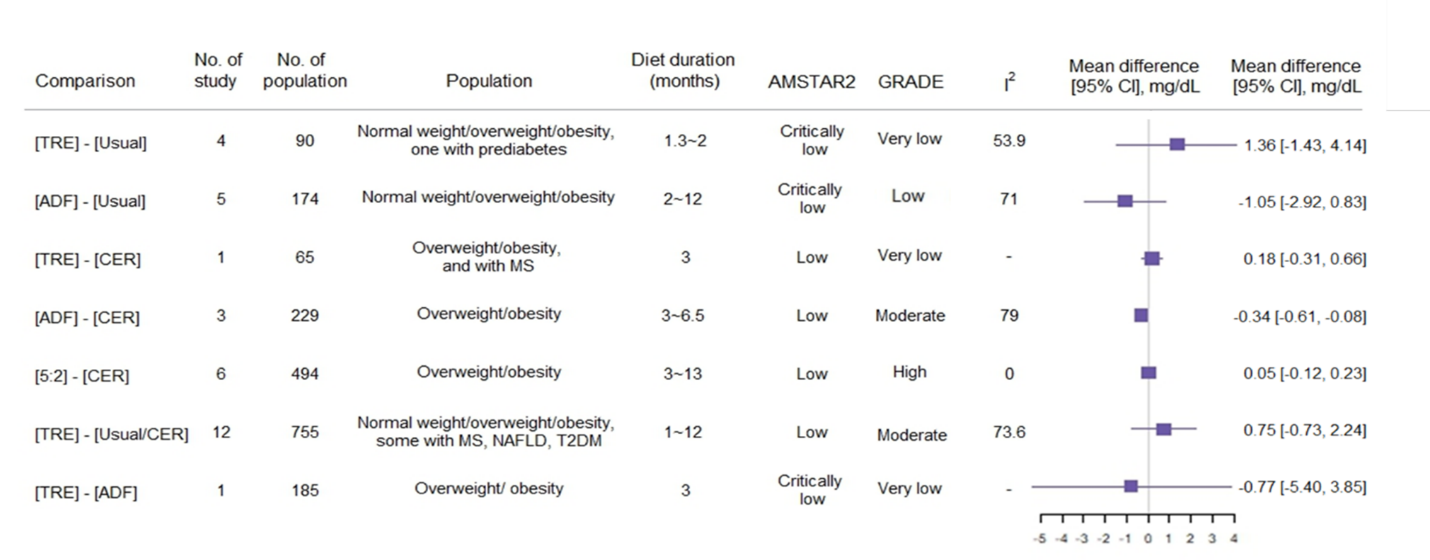


4:3: regular eating for four days and energy restriction for three days per week, 5:2: regular eating for five days and energy restriction for two days per week, ADF: alternate day fasting, CER: continuous energy restriction, MS: metabolic syndrome, NAFLD: non-alcoholic fatty liver disease, STIER: short-term intermittent energy restriction, T2DM: Type 2 Diabetes Mellitus, TRE: time-restricted eating

# Figure S2. Risk of bias in the original studies included in the systematic review


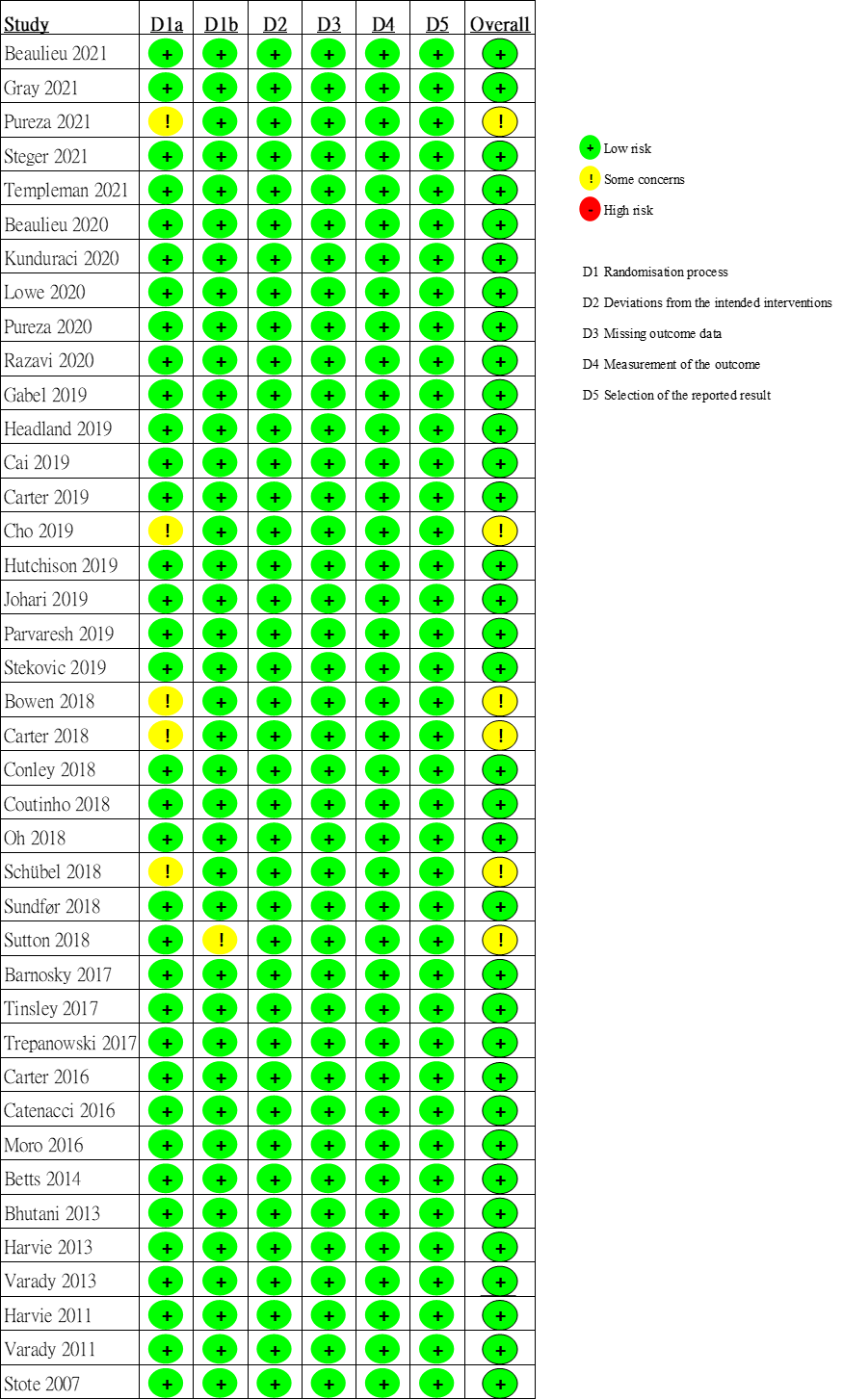


# Figure S3. Network plots

## (A) Body weight (BW)

## (B) Body mass index (BMI)

## (C) Fat mass (FM)

## (D) Fat free mass/Lean mass (FFM/LM)

## (E) Waist circumference (WC)

## (F) Systolic blood pressure (SBP)

## (G) Diastolic blood pressure (DBP)

## (H) Fasting blood glucose (FBG)

## (I) Fasting insulin (FI)

## (J) Hemoglobin A1c (HbA1c)

## (K) Triglycerides (TG)

## (L) Total cholesterol (TC)

## (M) Low density lipoprotein cholesterol (LDL-C)

## (N) High density lipoprotein cholesterol (HDL-C)

5:2: regular eating for five days and energy restriction for two days per week, ADF: alternate day fasting, CER: continuous energy restriction, TRE: time-restricted eating

# Figure S4. Results for surface under the cumulative ranking (SUCRA) analaysis

## (A) Body weight (BW)

## (B) Body mass index (BMI)

## (C) Fat mass (FM)

## (D) Fat free mass/Lean mass (FFM/LM)

## (E) Waist circumference (WC)

## (F) Systolic blood pressure (SBP)

## (G) Diastolic blood pressure (DBP)

## (H) Fasting blood glucose (FBG)

## (I) Fasting insulin (FI)

## (J) Hemoglobin A1c (HbA1c)

## (K) Triglycerides (TG)

## (L) Total cholesterol (TC)

## (M) Low density lipoprotein cholesterol (LDL-C)

## (N) High density lipoprotein cholesterol (HDL-C)

5:2: regular eating for five days and energy restriction for two days per week, ADF: alternate day fasting, CER: continuous energy restriction, TRE: time-restricted eating

# Figure S5. Results for the sensitivity analysis restricted to healthy people with surface under the cumulative ranking (SUCRA)


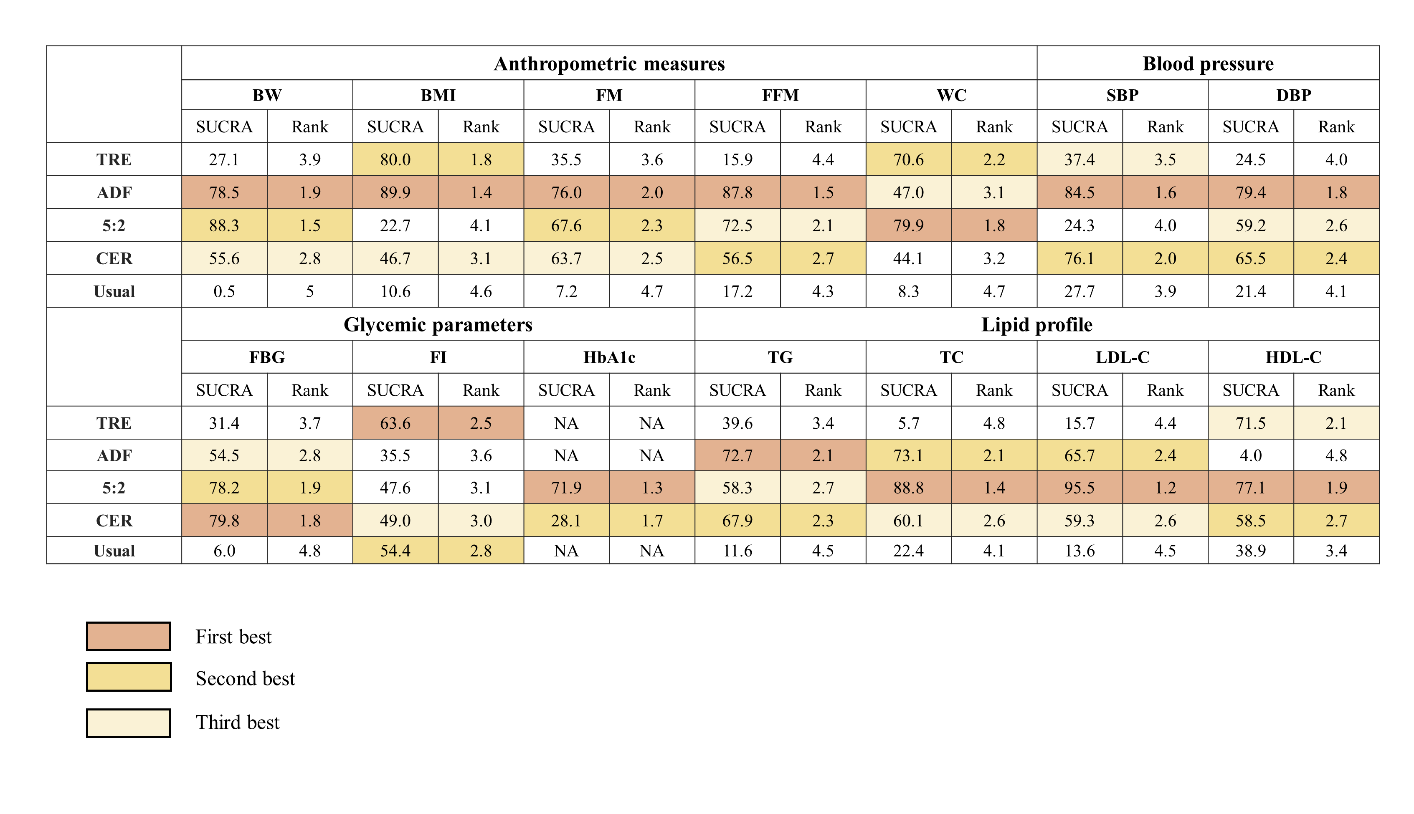


5:2: regular eating for five days and energy restriction for two days per week, ADF: alternate day fasting, BMI: body mass index, BW: body weight, CER: continuous energy restriction, DBP: diastolic blood pressure, FBG: fasting blood sugar, FFM: fat free mass, FI: fasting insulin, FM: fat mass, HbA1c: glycosylated hemoglobin A1c, HDL-C: high density lipoprotein cholesterol, LDL-C: low density lipoprotein cholesterol, SBP: systolic blood pressure, SUCRA: surface under the cumulative ranking curve, TC: Total cholesterol, TG: triglycerides, TRE: time-restricted eating, WC: waist circumference

Mixed strategy comparisons (5:2/4:3) and STIER (as it deviated from the standard regimen of IF) were excluded in this quantitative network meta-analysis.
